# Therapeutic Potential of the Deubiquitinase BRCC3 in Attenuating Ischemic Myocardial Injury and Ventricular Remodeling

**DOI:** 10.34133/research.1418

**Published:** 2026-09-03

**Authors:** Deshan Yao, Sichuan Wang, Rujun Li, Xin Pan, Zixuan Chen, Mengxuan Yuan, Pei Zhao, Zhengang Zhang, Kaizheng Gong

**Affiliations:** Department of Cardiology, Institute of Cardiovascular Disease, Yangzhou Key Lab of Innovation Frontiers in Cardiovascular Disease, Affiliated Hospital of Yangzhou University, Yangzhou University, Yangzhou, Jiangsu 225001, China.

## Abstract

The transforming growth factor-β1 (TGF-β1)/Smad3 pathway drives DNA damage, necrosis, and ventricular remodeling following acute myocardial infarction (AMI); however, effective therapies targeting this cascade remain limited. Here, we investigated the role of the deubiquitinating enzyme BRCA1/BRCA2-containing complex subunit 3 (BRCC3) in postinfarction cardiac remodeling and dysfunction using a mouse AMI model established via left coronary artery ligation. The cardiac structure and function were evaluated by echocardiography, Western blotting, and histomorphometry. We found that cardiac BRCC3 expression was markedly down-regulated after AMI. Cardiac-specific BRCC3 overexpression inhibited TGF-β1/Smad3 activation, reduced the infarct size, attenuated adverse remodeling and DNA double-strand breaks, improved cardiac function, and enhanced survival, whereas cardiac-specific *Brcc3* gene knockout exacerbated these phenotypes. In vitro, BRCC3 suppressed TGF-β1-induced hypertrophic gene expression and DNA damage in cardiomyocytes, as well as profibrotic responses in cardiac fibroblasts. Mechanistically, BRCC3 bound to the MH2 domain of Smad3 and removed K63-linked polyubiquitination at lysine 333 (K333), thereby inhibiting Smad3 phosphorylation at Ser423/425. Collectively, these findings demonstrate that BRCC3 protects against ischemic injury and pathological remodeling by deubiquitinating Smad3 at K333 to antagonize TGF-β1/Smad3 signaling, identifying BRCC3 as a potential therapeutic target for preventing post-AMI heart failure.

## Introduction

In response to acute myocardial ischemia, excessive activation of the transforming growth factor-β1 (TGF-β1)/Smad2,3 pathway contributes to cellular DNA damage, necrosis, tissue repair, and ventricular remodeling and dysfunction [1,2]. In the myocardium, TGF-β1 is secreted by cardiomyocytes and cardiac fibroblasts (CFs) under various stresses [3–5]. Generally, bioactive TGF-β1 binds to type I and type II TGF-β receptors to activate both Smad2 and Smad3 cascades. Although Smad2 and Smad3 share structural and biochemical similarities, accumulating evidence indicates that Smad3 plays distinct roles from Smad2 in regulating CF and macrophage phenotype and function within the infarcted myocardium [6,7]. For instance, mice with fibroblast-specific Smad3 loss exhibit exacerbated adverse cardiac remodeling following reperfused infarction and an increased incidence of late rupture following nonreperfused infarction. Conversely, cardiomyocyte-specific Smad3 deletion reduces cardiomyocyte apoptosis and attenuates post-myocardial infarction (post-MI) remodeling and dysfunction [4].

Smad3 activation is governed by several mechanisms, including posttranslational modifications. Classically, phosphorylation at Ser423/425 within the C-terminal SSXS motif by the type I TGF-β receptor is essential for Smad3 interaction with Smad4, nuclear translocation, and transactivation [8]. Smad ubiquitination regulatory factor 2 (Smurf2)-mediated dual monoubiquitination of Smad3 inhibits the formation of Smad3–Smad4 complexes [9]. Additionally, the E3 ubiquitin ligase complex ROC1–SCF (Fbw1a), mediated by RNF111, induces K48-linked ubiquitination of Smad3, facilitating its proteasomal degradation in a ligand-dependent manner [10]. Unlike K48-linked polyubiquitination, K63-linked polyubiquitination primarily regulates intracellular protein trafficking [11,12], transcription [13], DNA repair [14], and kinase signaling [15].

BRCA1/BRCA2-containing complex subunit 3 (BRCC3) is a zinc metalloprotease belonging to the JAMM/MPN^+^ family of deubiquitinases (DUBs) that specifically cleaves K63-linked polyubiquitin chains [16,17]. Initially identified as a core component of the BRCA1-A complex, BRCC3 mediates homologous recombination to repair DNA double-strand breaks (DSBs) [18]. Emerging evidence suggests that BRCC3 participates in diverse cellular processes, including autophagy [19], NLRP3 inflammasome activation [20], and development [21]. In the cardiovascular system, *Brcc3* morphant zebra fish exhibit angiogenesis defects, and loss-of-function *Brcc3* mutations are associated with moyamoya angiopathy [22]. Using co-immunoprecipitation (co-IP) coupled with liquid chromatography–tandem mass spectrometry (LC–MS/MS) analysis, we previously identified BRCC3 as an Smad3-interacting protein in rat pulmonary artery smooth muscle cells [23]. More recently, we demonstrated that BRCC3 overexpression activates the BMP2–ALK2–Smad1/5 pathway, thereby preventing hypoxia-induced pulmonary vascular remodeling and hypertension [17]. Studies have shown that the ablation of ABRO1—a chaperone protein that partners with BRCC3 in the BRCA1-A complex—exacerbates oxidative stress injury and cardiomyocyte death following myocardial ischemia [24]. However, despite its critical role in this complex, whether BRCC3 exerts a protective effect against pathological processes associated with myocardial ischemia remains unknown.

Although the link between the prolonged activation of TGF-β1–Smad2,3 signaling and cardiac remodeling and dysfunction is well established, effective clinical strategies targeting this cascade in MI remain lacking. We hypothesized that BRCC3 functions as a specific DUB to regulate Smad3 K63-linked polyubiquitination, thereby interfering with the activated TGF-β–Smad3 cascade and mitigating ventricular remodeling post-acute myocardial infarction (post-AMI). In this study, we demonstrate that BRCC3 removes K63-linked polyubiquitination of Smad3 at Lys333, antagonizing ischemia-induced TGF-β signaling and subsequent DNA DSB formation by enhancing myocardial DNA damage repair. Clinically, we observed reduced BRCC3 levels in both cardiac tissues and serum from patients with ischemic heart disease and chronic heart failure compared to those in controls. These findings suggest that BRCC3 serves as an endogenous protective factor against ischemia-induced cardiac remodeling and represents a promising therapeutic target for preventing post-MI heart failure.

## Results

### BRCC3 is decreased in the ischemic myocardium

**Fig. 1.**
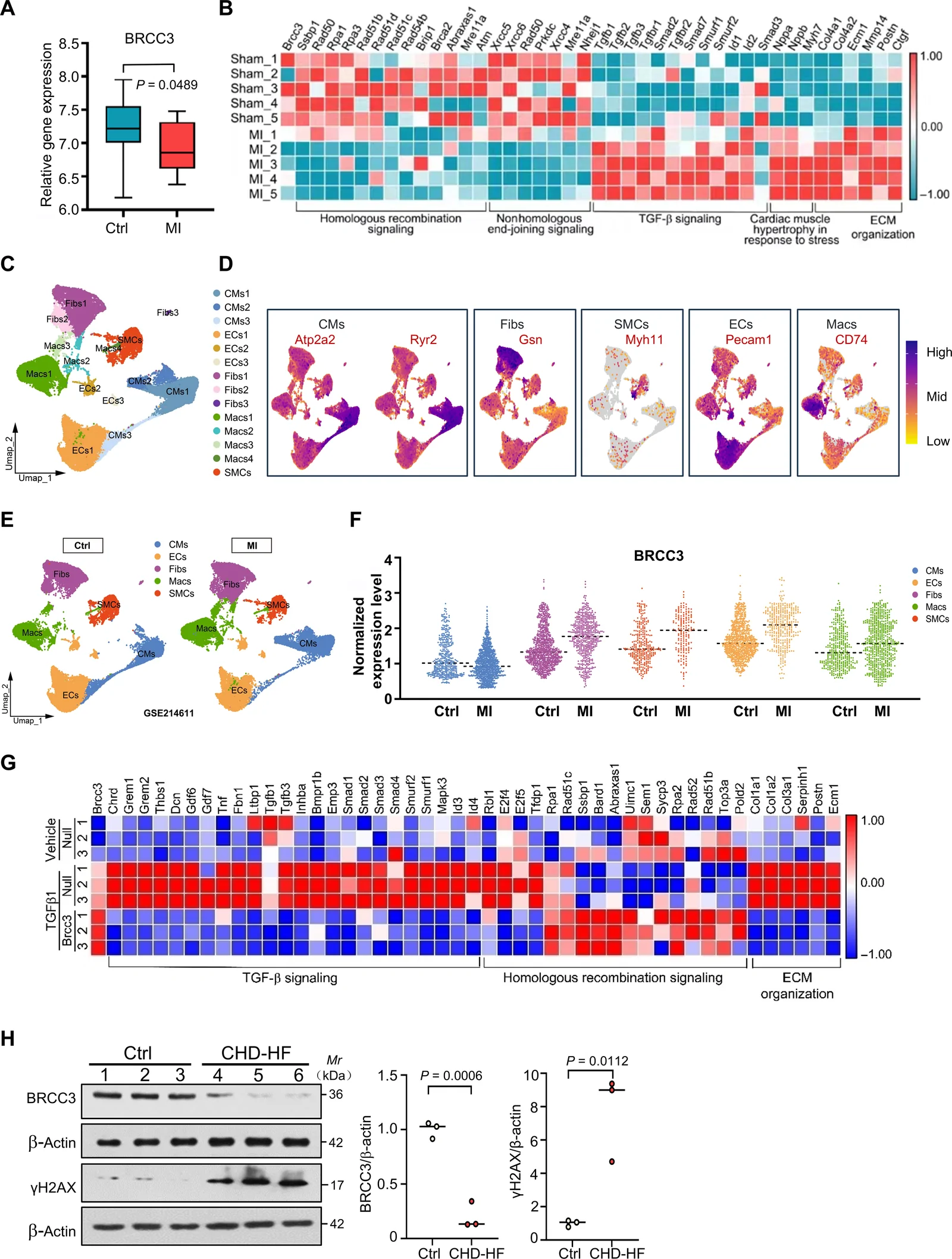
Ischemic stress led to myocardial BRCA1/BRCA2-containing complex subunit 3 (BRCC3) down-regulation in humans and mice. (A) RNA sequencing (RNA-seq) data mining of BRCC3 messenger RNA (mRNA) in patients with myocardial infarction (MI). The box plot shows the expression of BRCC3 mRNA in myocardial samples from patients with and without MI (*n* = 5 and *n* = 22 [Ctrl]). Data were from a Gene Expression Omnibus (GEO) RNA-seq dataset (GSE48060). (B) Heatmap showing the expression levels of the indicated genes in left ventricular myocardial samples from MI mice (*n* = 4) and sham-operated mice (*n* = 5). Data were from a GEO RNA-seq dataset (GSE153485). (C) Single-nucleus RNA sequencing (snRNA-seq) data were obtained from GEO with accession number GSE214611. Uniform manifold approximation and projection plot showing heart nuclei from 8 mice with clusters labeled by cell type. (D) Expression of selected specific marker genes correlated with cardiomyocytes (CMs), cardiac fibroblasts (Fibs), smooth muscle cells (SMCs), endothelial cells (ECs), and macrophage (Macs). (E) Uniform manifold approximation and projection plot showing heart nuclei from 8 mice in normal (Ctrl) and myocardial infarction groups with clusters labeled by cell type. (F) Comparison of normalized BRCC3 expression level (filtering out *Brcc3* = 0 cells) in 5 clusters between the control and MI groups. (G) Heatmaps showing differentially expressed genes involved in transforming growth factor-β (TGF-β) signaling pathways (25 differentially expressed genes), homologous recombination signaling (15 differentially expressed genes), and extracellular matrix remodeling (6 differentially expressed genes) in cultured cardiomyocytes transfected with AD-Null vs AD-*Brcc3* and treated with the vehicle or TGF-β1. (H) Western blot analysis of BRCC3 and γH2AX protein levels in the atrial appendage tissues of coronary artery disease patients with normal heart function (Ctrl) and heart failure (CHD-HF) (*n*= 3). Data are mean ± standard error of the mean (SEM) (a Student *t* test was used for comparisons between 2 groups).

To assess the role of BRCC3 in the heart, we first analyzed a Gene Expression Omnibus (GEO) RNA sequencing (RNA-seq) dataset (GSE48060) comparing cardiac transcriptomes from patients with and without MI (*n* = 5 and *n* = 22, respectively). The BRCC3 messenger RNA (mRNA) levels in left ventricles tended to be lower in patients with MI than in those without (Fig. 1A). We further analyzed the GEO RNA-seq dataset (GSE153485) generated from MI versus sham-operated mice (*n* = 5 in each group). Consistent with human data, the left ventricular myocardial mRNA levels of BRCC3 were also decreased 24 h after surgery in MI mice. Along with reduced BRCC3 levels, genes involved in homologous recombination (e.g., *Rad51a*, *Atm*, and *Mre11a*) and nonhomologous recombination (e.g., *Rad50*) were down-regulated. Conversely, the levels of genes involved in TGF-β signaling (e.g., *Tgfb*, *Smurf1,2*, and *Smad2*), cardiac hypertrophy (e.g., *Nppa* and *Myh7*), and extracellular matrix remodeling (e.g., *Postn* and *Ctgf*) were up-regulated in MI mice compared with those in sham controls (Fig. 1B), suggesting that ischemia injury triggers the activation of myocardial DNA damage repair mechanisms and remodeling-related TGF-β signaling pathway.

To further clarify the expression profile of BRCC3 in different cell types in an infarcted heart, we scanned a single-nucleus RNA-seq dataset of mouse heart (GSE214611) derived from a sham surgery group (Ctrl, *n* = 3) and a left anterior descending artery (LAD) ligation-induced AMI model group (MI, *n* = 5) [25]. Based on the specific marker gene, a total of 14 cell clusters were further classified into 5 major cell types (Fig. 1C and D). BRCC3 is widely expressed in various cell types throughout the body. Unlike these in myocardial fibroblasts, smooth muscle cells, macrophages, and endothelial cells, BRCC3 expression in cardiomyocytes was down-regulated in the MI group compared with that in the control group 7 d after surgery (Fig. 1E and F). This finding suggests that BRCC3 plays a more critical role in regulating the fate of myocardial cells.

To directly further explore the above bioinformatics analytic results involving the potential association between BRCC3 and the TGF-β signaling pathway, we transfected cardiomyocytes with adenovirus AD-*Brcc3* or Ad-Ctrl for 3 d and then treated them with the vehicle or TGF-β1 for 24 h to examine the BRCC3-regulated transcriptome. Database for Annotation, Visualization and Integrated Discovery (DAVID) analysis showed that genes involved in several cardiac remodeling-related pathways, e.g., DNA damage repairment (GO:0006974), cardiac hypertrophy (GO:0014898), positive regulation of fibroblast proliferation (GO:0048146), extracellular matrix remodeling (GO:0030198), and TGF-β signaling pathway (map04350, Kyoto Encyclopedia of Genes and Genomes), were affected by BRCC3 overexpression. A total of 25,777 genes were detected in the RNA-seq, and the differentially expressed genes were analyzed. The corresponding heatmap in Fig. 1G demonstrates the up-regulation of genes in the TGF-β signaling pathway, such as *Smad2*, *Smad3*, *Smurf2*, *Fbn1*, and *Tgfb1*, and extracellular matrix organization, including the *Coll1a1*, *Coll3a1*, and *Postn* genes, was suppressed by BRCC3. Also, homologous-recombination-related genes in response to TGF-β1 treatment, such as *Rad51c*, *Abraxas1*, and *E2f4*, were regulated by BRCC3 overexpression.

Clinically, we also tested the level of BRCC3 expression in the heart tissues of patients with coronary heart disease with or without heart failure. The baseline characteristics of these patients are shown in Table S1. We observed that with the decline in heart function in patients, the expression of BRCC3 was down-regulated and γH2AX was up-regulated compared with subjects with preserved heart function (Ctrl) (Fig. 1H), indicating that the failing myocardium may be accompanied by down-regulation of BRCC3, sustained activation of the TGF-β pathway, and DNA damage, suggesting that BRCC3 down-regulation may be associated with the severity of cardiac dysfunction.

### BRCC3 elicits cardioprotection against acute ischemic stress

**Fig. 2.**
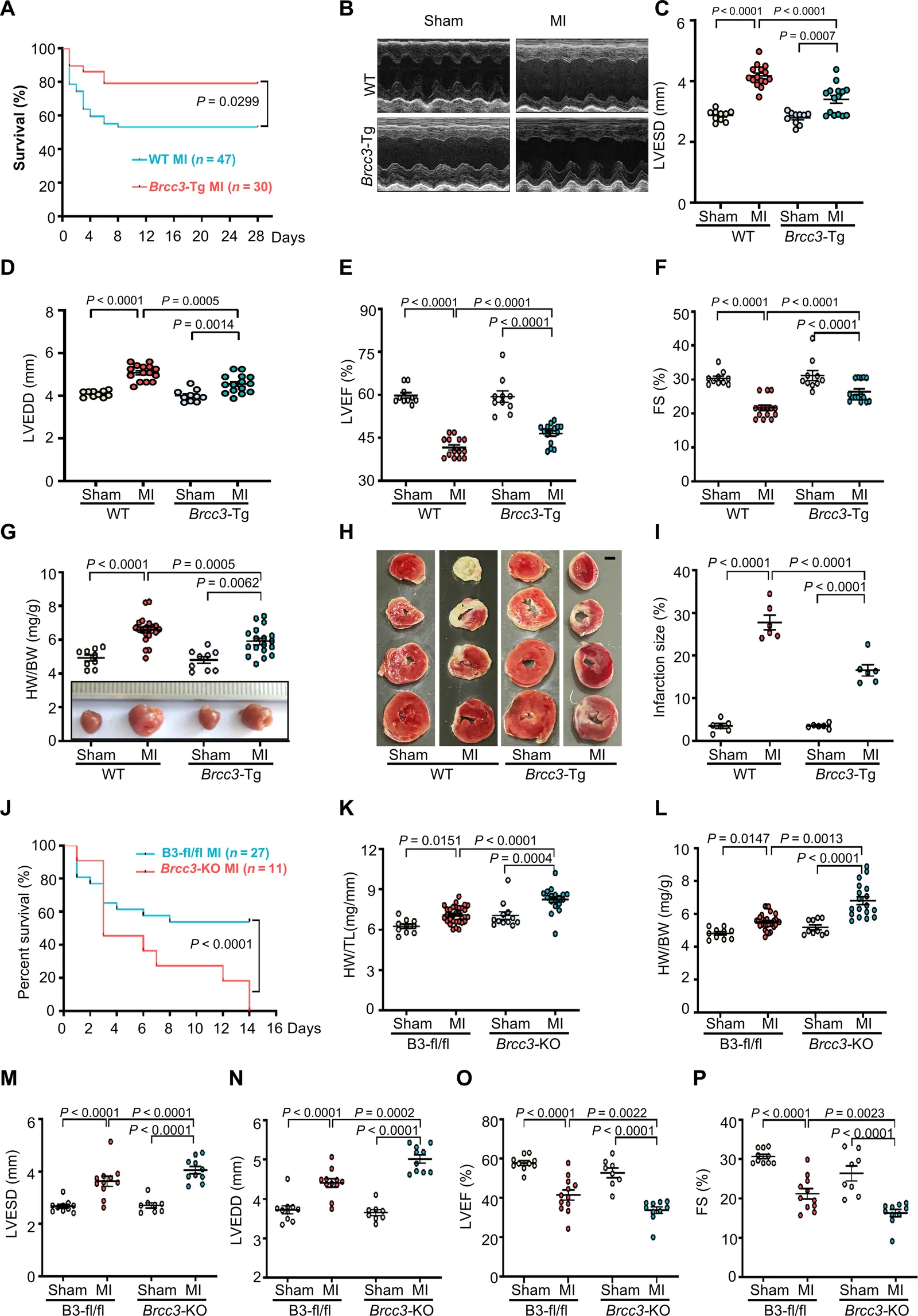
Cardiac BRCA1/BRCA2-containing complex subunit 3 (BRCC3) overexpression or knockout in mice affects survival, infarct size, and cardiac function. (A) Wild-type (WT) and *Brcc3*-Tg mice underwent left anterior descending artery (LAD) ligation or sham surgery. Survival was monitored on day 28 after myocardial infarction (MI). (B to F) Left ventricular structure and function were determined by echocardiographic study using a Vevo 3100 machine with an MX550S transducer 110 (Visual Sonics, Toronto, Canada) in WT or *Brcc3*-Tg mice with or without MI 28 d after surgery (WT sham, *n* = 10; WT MI, *n* = 15; *Brcc3*-Tg sham, *n* = 10; *Brcc3*-Tg MI, *n* = 15 mice). Data are mean ± SEM; one-way analysis of variance (ANOVA) followed by Bonferroni multiple comparison tests. (G) Representative photograph of hearts on day 28 after MI. Whole hearts were weighed and the ratio of heart weight to body weight (HW/BW) is shown. (H) Representative areas of MI in *Brcc3*-Tg and WT groups revealed by 2,3,5-triphenyl tetrazolium chloride (TTC) staining. Scale bar = 1 mm. (I) The infarction size was averaged from 6 mice in each group. (J) *Brcc3*-KO and the littermate *Brcc3*^flox+^/Cre^−^ control (B3-fl/fl) mice underwent LAD ligation or sham surgery. Survival was monitored in B3-fl/fl and *Brcc3*-KO mice at day 14 after MI. (K and L) Whole hearts were weighed and the ratios of heart weight to tibia length (HW/TL) and body weight (HW/BW) were calculated. (M to P) The left ventricular end-systolic diameter (LVESD), left ventricular end-diastolic diameter (LVEDD), left ventricular fractional shortening (LVFS), and left ventricular ejection fraction (LVEF) were determined at 7 d after sham or MI surgery by M-mode echocardiography. *n* = 8 to 12 each group. Data are mean ± SEM (one-way ANOVA followed by Bonferroni multiple comparison tests).

Since we show that *Brcc3* expression was decreased in cardiomyocytes compared with other cells in the heart after MI, we generated mice with cardiac-specific BRCC3 overexpression (*Brcc3*-Tg) and knockout (*Brcc3*-KO) to examine the involvement of BRCC3 in MI. Neither *Brcc3*-Tg nor *Brcc3*-KO neonates were born with noticeable cardiac defects or alterations in heart rate and blood pressure measured by a noninvasive tail-cuff system under unstressed conditions. LAD ligation was performed to induce AMI in these 2 lines of mice and their respective littermate controls. The post-MI survival rate was markedly improved in *Brcc3*-Tg mice compared with that in wild-type (WT) controls (Fig. 2A). Echocardiographic studies revealed that the left ventricular end-systolic diameter (LVESD) and left ventricular end-diastolic diameter (LVEDD) were reduced, while fractional shortening (FS) and the left ventricular ejection fraction (LVEF) showed an increase in *Brcc3*-Tg mice compared with those in WT controls (Fig. 2B to F). Similarly, the ratio of heart weight to body weight (HW/BW) in *Brcc3*-Tg mice was decreased (Fig. 2G), and the ratio of heart weight to tibia length (HW/TL) also decreased markedly (7.23 vs 6.23). In our preliminary experiment, Evans blue staining of the myocardium 3 d after AMI showed no significant difference in the area at risk between the *Brcc3*-Tg group and the WT control group. However, the myocardial infarct size at 4 weeks after MI was smaller in *Brcc3*-Tg than in WT mice (Fig. 2H and I). By contrast, *Brcc3*-KO mice showed lower post-MI survival due to pulmonary edema or heart rupture (Fig. 2J), an enlarged infarct size (Fig. S2), and increased ratios of HW/TL, HW/BW, LVEDD, and LVESD but decreased FS and LVEF, compared with littermate *Brcc3*^flox+^/Cre^−^ MI mice (B3-fl/fl MI) (Fig. 2K to P). Together, the results from the *Brcc3* gain- and loss-of-function mice demonstrate the protective role of BRCC3 against cardiac stress imposed by MI.

### BRCC3 alleviates cardiac fibrosis in MI mice by antagonizing the activated TGF-β–Smad3 pathway

**Fig. 3.**
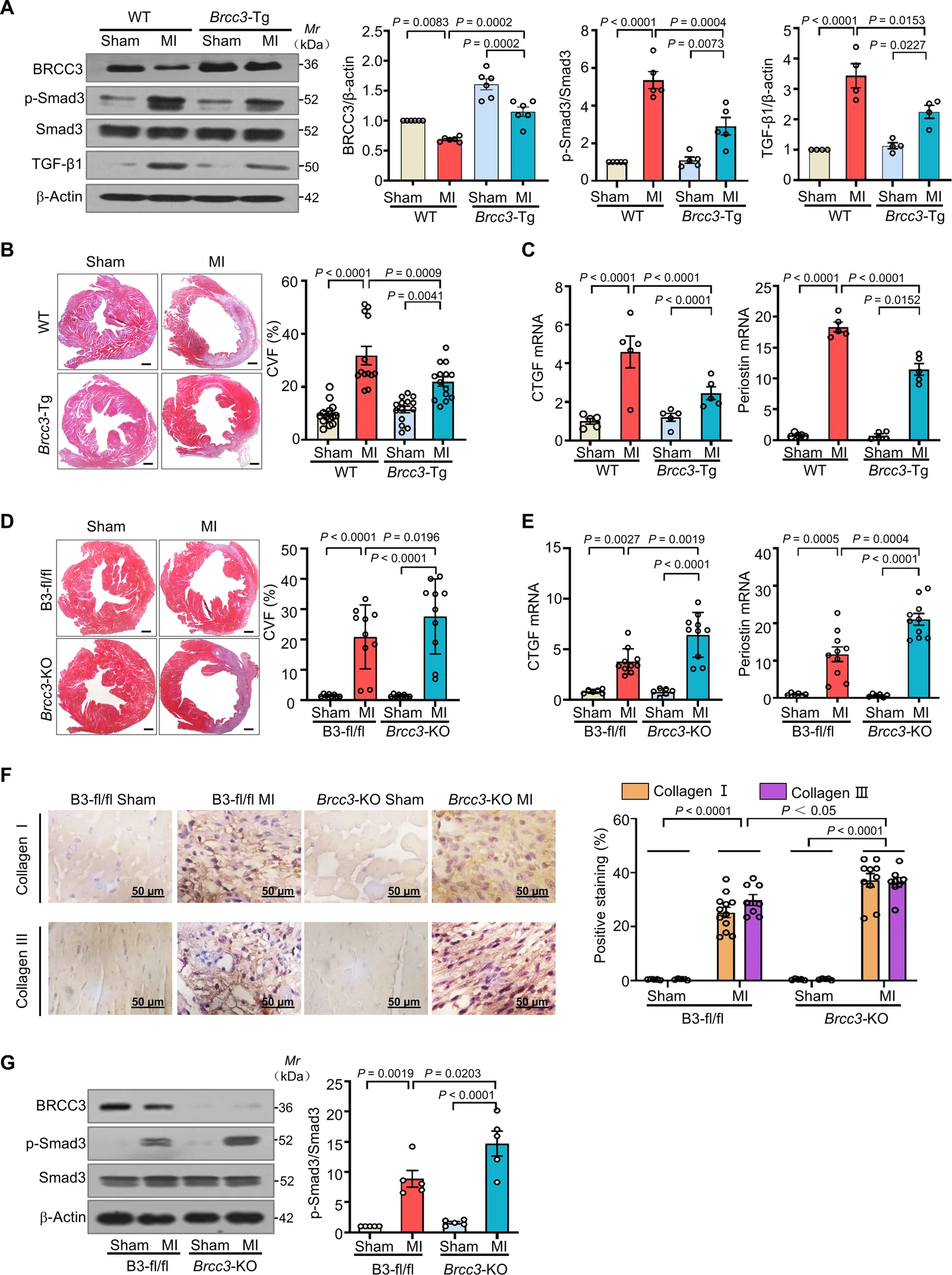
BRCA1/BRCA2-containing complex subunit 3 (BRCC3) suppresses myocardial infarction (MI)-induced cardiac fibrosis in mice. Wild-type (WT), *Brcc3*-Tg, B3-fl/fl, and *Brcc3*-KO mice were subjected to left anterior descending artery (LAD) ligation or sham surgery. (A) Representative Western blot analysis of the BRCC3, transforming growth factor-β1 (TGF-β1), Smad3, and phosphorylated Smad3 (p-Smad3) levels in the hearts of WT and *Brcc3*-Tg mice (*n* = 7 to 8 mice/group). β-Actin was a loading control. (B) Representative Masson-stained light micrographs of heart sections at 40× magnification and the myocardial collagen volume fraction (CVF) in WT and *Brcc3*-Tg mice after MI. Scale bar = 500 μm. (C) Real-time reverse transcription polymerase chain reaction (RT-PCR) analysis of the messenger RNA (mRNA) levels of *CTGF* and *Periostin* normalized to the *GADPH* level in the left ventricle (LV) from WT and *Brcc3*-Tg mice with or without MI. (D) Representative Masson-stained light micrographs of heart sections at 40× magnification and the myocardial CVF in B3-fl/fl and *Brcc3*-KO mice after MI. Scale bar = 500 μm. (E) Real-time RT-PCR analysis of the mRNA levels of *CTGF* and *Periostin* normalized to the *GADPH* level in LV from B3-fl/fl and *Brcc3*-KO mice after MI. (F) Immunohistochemical staining (200× magnification) for myocardial collagen I/III in B3-fl/fl and *Brcc3*-KO mice after MI was analyzed. (G) Western blot analysis for the LV levels of BRCC3, p-Smad3, and Smad3 protein expression. β-Actin was a loading control. Data are mean ± SEM (one-way ANOVA followed by Bonferroni multiple comparison tests).

We next studied the mechanism by which BRCC3 protected mouse hearts at the molecular and tissue levels. Although the result from the GEO RNA-seq dataset analysis showed that AMI did not induce an increase in *Smad3* mRNA expression in infarcted mouse myocardium, compared with Smad2 and Smad3 target genes, such as *Postn* and *Ctgf* (Fig. 1B), our Western blot analysis showed that MI stress not only increased the expression of TGF-β and phosphorylated Smad3 (Ser-423/425), which indicates TGF-β pathway activation, but also reduced the BRCC3 level in the myocardium of WT mice. Interestingly, cardiac-specific overexpression of BRCC3 in *Brcc3*-Tg mice rectified TGF-β1 and Smad3 activation in the myocardium (Fig. 3A), indicating an inhibitory effect of BRCC3 on the activated TGF-β1–Smad3 signal. Phenotypically, consistent with the findings of previous studies [26,27], collagen fiber staining (Masson, collagen type I, and collagen type III) in the heart tissue of mice from the sham operation group yielded negative results. MI increased cardiac fibrosis in the ischemic area of WT mice, as revealed by augmented collagen volume fraction (CVF) in Masson’s trichrome staining and increased expression of the extracellular matrix molecules connective tissue growth factor (CTGF) and periostin. Of note, MI-induced cardiac fibrosis was significantly reduced in *Brcc3*-Tg mice (Fig. 3B and C). By contrast, the cardiac ablation of BRCC3 potentiated MI-induced increases in myocardial CVF, expression of *CTGF* and *Periostin*, collagen I/III deposition, and excessive phosphorylation of Smad3 compared with those in littermate B3-fl/fl mice (Fig. 3D to G). Cardiomyocytes account for approximately 80% of the heart’s volume and are the main cells that make up the heart. Similar to previous studies, BRCC3 is almost not expressed in the myocardial tissue of mice with cardiomyocyte-specific *Brcc3* knockout. Together, these results suggest that BRCC3 prevents MI-induced cardiac fibrosis and preserves heart function by inhibiting excessive TGF-β–Smad3 activation in the infarcted myocardium.

### BRCC3 inhibits TGF-β1 signaling in cardiomyocytes and CFs

**Fig. 4.**
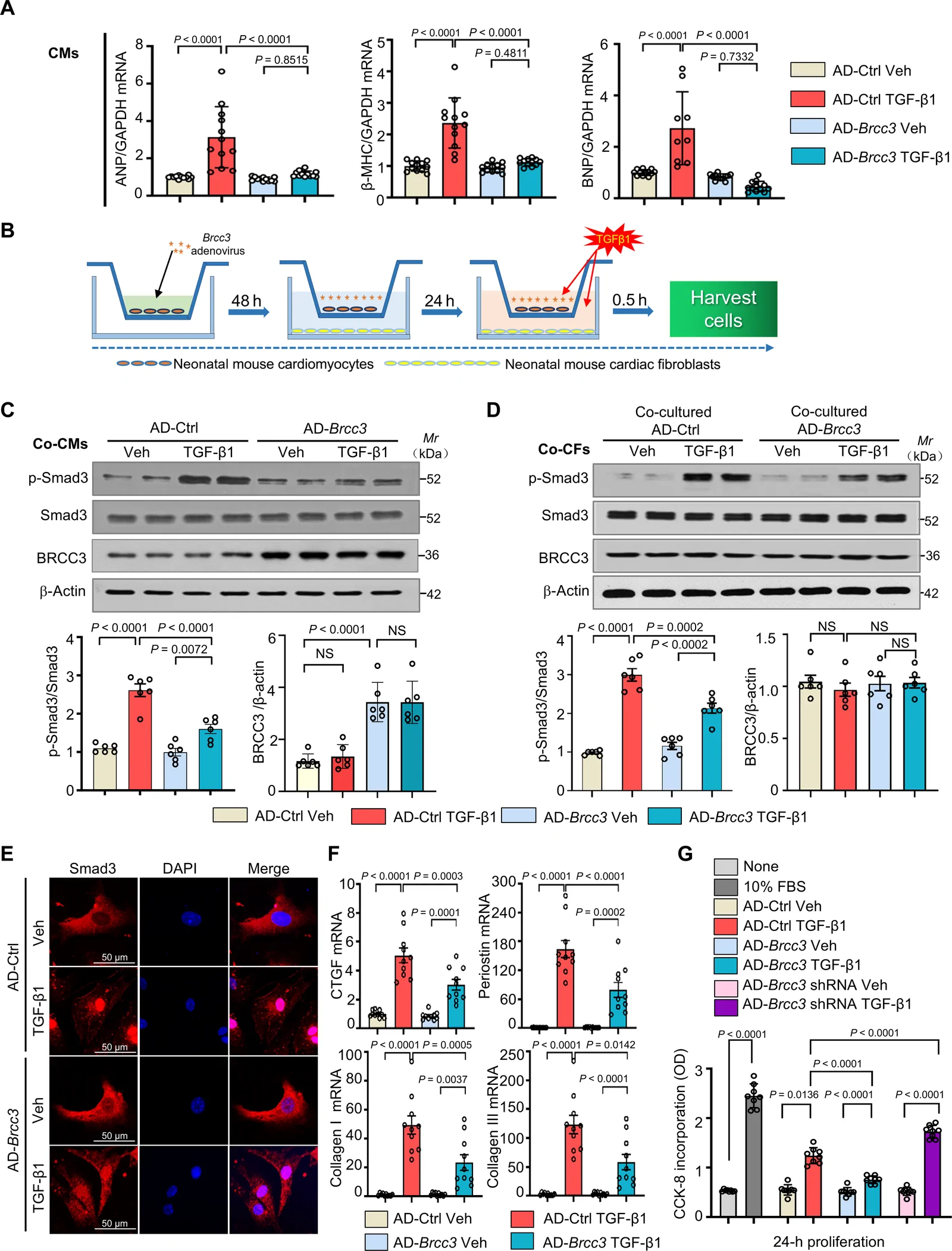
BRCA1/BRCA2-containing complex subunit 3 (BRCC3) inhibits transforming growth factor-β1 (TGF-β1)-induced cardiac hypertrophy and fibrosis in vitro. (A) Neonatal mouse cardiomyocytes (CMs) were isolated and transfected with an adenovirus overexpressing *Brcc3* (AD-*Brcc3*) or an empty vector (AD-Ctrl) and then treated with TGF-β1 (4 ng/ml) or the vehicle for 24 h. Real-time reverse transcription polymerase chain reaction (RT-PCR) analysis of the messenger RNA (mRNA) levels of cardiac-hypertrophy-related genes, including *ANP*, *β-MHC*, and *BNP*, normalized to *GADPH* level. (B) Schematic diagram of the co-culture systems including CMs (red) infected with AD-*Brcc3* and cardiac fibroblasts (CFs; yellow). Neonatal mouse CMs were infected with AD-*Brcc3* or AD-Ctrl and then co-cultured with CFs for 24 h. All cells were challenged with TGF-β1 (4 ng/ml) or the vehicle for 30 min (*n* = 6). (C and D) Western blot analysis of the protein levels of p-Smad3, Smad3, and BRCC3 in CMs and co-cultured CFs. β-actin was a loading control. (E) Representative immunofluorescence staining (scale bar = 50 μm) of Smad3 (red) in CFs infected with AD-*Brcc3* or AD-Ctrl in response to TGF-β1 treatment for 1 h. Nuclei were counterstained with 4′,6-diamidino-2-phenylindole (DAPI; blue). (F) Real-time RT-PCR analysis of the mRNA levels of extracellular matrix molecules including *CTGF*, *periostin*, and *Collagen I* and *III* in CFs treated with TGF-β1 (4 ng/ml) or the vehicle for 12 h. Glyceraldehyde-3-phosphate dehydrogenase (GAPDH) was the internal control. (G) CFs were infected AD-*Brcc3*, AD-*Brcc3* short hairpin RNA (shRNA), or AD-Ctrl for 72 h and then treated with TGF-β1 (4 ng/ml) or the vehicle (Veh) for 24 h. The proliferation of CFs was measured using Cell Counting Kit-8 (CCK-8); 10% fetal bovine serum (FBS) was the positive control (*n* = 8). Results are expressed as mean ± SEM. Statistical analysis: One-way ANOVA followed by Bonferroni multiple comparison tests. NS, nonsignificant.

Next, we used cultured cardiomyocytes and CFs with BRCC3 gain of function to explore the cellular mechanism by which BRCC3 protects the heart against ischemia-induced cardiac remodeling. Consistent with the results from the above in vivo study, we found that adenovirus-mediated BRCC3 overexpression in neonatal cardiomyocytes inhibited the TGF-β1-increased hypertrophic gene expression, i.e., *ANP*, *β-MHC*, and *BNP* (Fig. 4A). During the onset of cardiac stress due to MI, cardiomyocytes increase their secretion of TGF-β1. The ensuing TGF-β1-stimulated plasticity of CFs contributes to cardiac fibrosis [28]. Thus, to understand why specific overexpression of BRCC3 in cardiomyocytes mediated by the α-MHC promoter in *Brcc3*-Tg mice significantly suppressed cardiac fibrosis, we tested the potential cross talk between cardiomyocytes and CFs with BRCC3 overexpression. Mouse cardiomyocytes infected with adenovirus AD-*Brcc3* or AD-Ctrl for 48 h were co-cultured with CFs for 24 h and then treated with TGF-β1 or the vehicle for 30 min (Fig. 4B). Not only did the overexpression of BRCC3 markedly inhibit TGF-β1-induced Smad3 phosphorylation in cardiomyocytes, but also TGF-β1-induced Smad3 activation was substantially attenuated in co-cultured CFs without BRCC3 overexpression (Fig. 4C and D). Similarly, adenovirus-mediated BRCC3 overexpression in CFs effectively inhibited TGF-β1-induced Smad3 nuclear translocation and the expression of *CTGF*, *Periostin*, and *collagen I/III* mRNA (Fig. 4E and F). Pretreatment with the specific Smad3 inhibitor (E)-SIS3 (10 μM) not only attenuated TGF-β1-induced mRNA expression of *CTGF* and *Periostin* but also fully counteracted BRCC3-mediated inhibitory action on extracellular matrix expression in CFs. To verify the underlying mechanism of the cross talk between the 2 cell types, we also collected the culture medium of co-cultured cells at 1 h, at 24 h, and after 0.5 h of TGF-β1 stimulation. We detected the expression of TGF-β1, interleukin-6 (IL-6), and extracellular vesicle milk fat globule-epidermal growth factor 8 (MFGE8) in the culture medium using enzyme-linked immunosorbent assay. The results showed that the overexpression of BRCC3 markedly inhibited the expression of TGF-β1 and IL-6 in the culture medium while up-regulating the expression of extracellular vesicle MFGE8. Taken together, the results suggest that overexpression of BRCC3 in cardiomyocytes affects fibroblast function through paracrine pathways (Fig. S3A to C).

Of note, the overexpression of BRCC3 had little effect on TGF-β1-induced Smad2 phosphorylation (Fig. S4A and B), suggesting that Smad3, but not Smad2, functions as a key target to mediate the antifibrotic effect of BRCC3. In addition, Cell Counting Kit-8 analysis showed that BRCC3 overexpression effectively suppressed, while BRCC3 knockdown increased, TGF-β1-induced the proliferation of CFs compared with their respective controls (Fig. 4G). These results suggest that BRCC3 antagonizes TGF-β1-induced Smad3, but not Smad2, activation in cardiac remodeling.

### BRCC3 removes specific K63-linked polyubiquitination of Smad3 at the lysine 333 residue

**Fig. 5.**
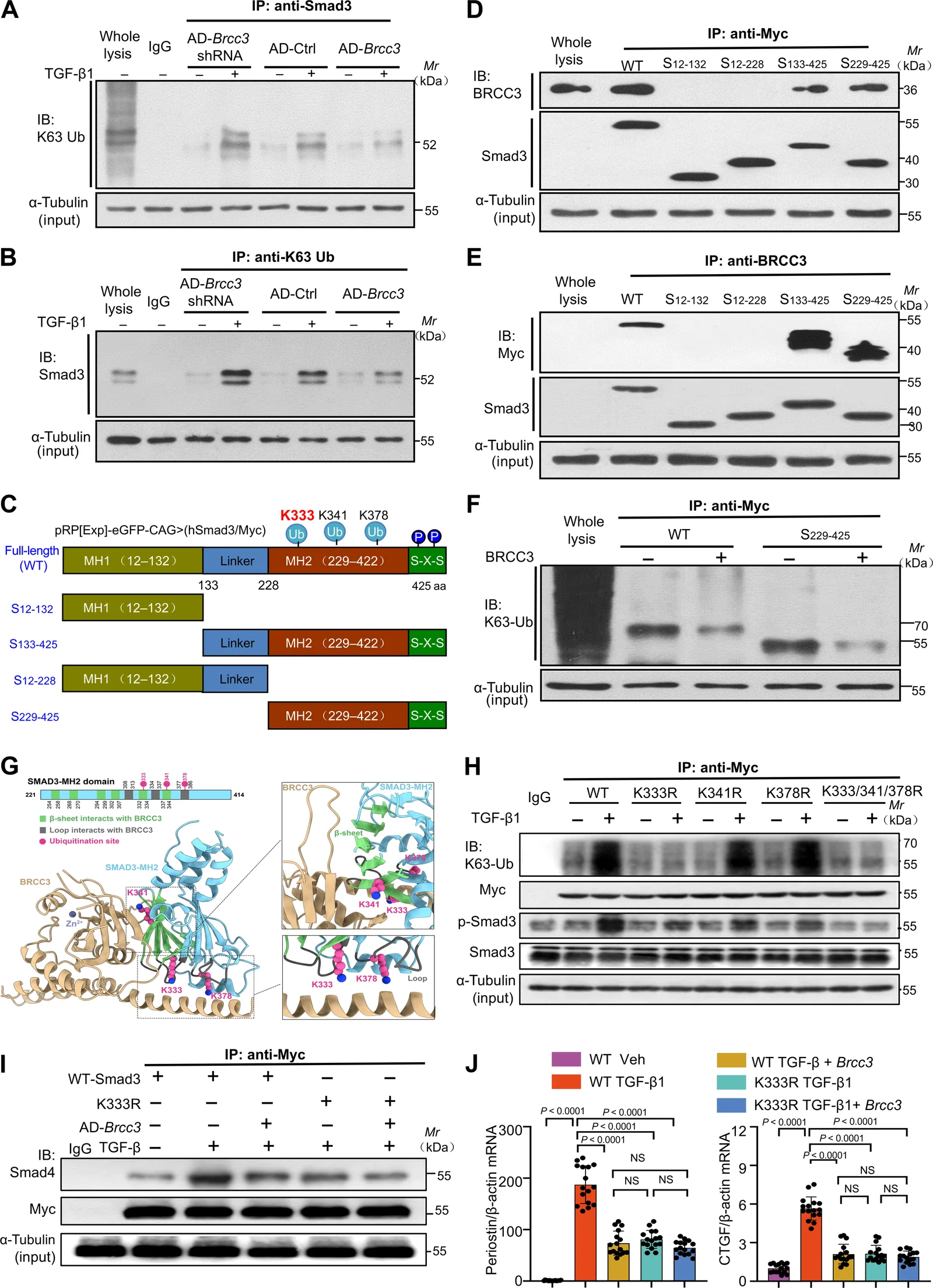
BRCA1/BRCA2-containing complex subunit 3 (BRCC3) decreases K63-linked polyubiquitination of Smad3. Adult mouse cardiac fibroblasts (CFs) were infected with AD-Ctrl or AD-*Brcc3* and then treated with transforming growth factor-β1 (TGF-β1; 4 ng/ml) or the vehicle for 30 min. (A and B) Cellular extracts were immunoprecipitated with anti-Smad3 or anti-K63-Ub. Immunoprecipitation with rabbit immunoglobulin G (IgG) antibody was the negative control. The immunoprecipitates were then subjected to immunoblotting with anti-K63-Ub or anti-Smad3 antibodies. (C) Schematic diagram of the full-length Smad3 (wild type, WT) plasmid constructs containing different domains and tagged with Myc. Three potential K63-linked polyubiquitination sites (i.e., K333, K341, and K378) in the Smad3 MH2 domain were predicted by using the online UbiqSite software. (D and E) Serum-starved HEK293 cells were transfected with 3 ng of various Smad3 variants for 72 h. Cellular extracts were immunoprecipitated with anti-Myc or anti-BRCC3 antibody. The immunoprecipitates were then subjected to immunoblotting analysis with anti-BRCC3 or anti-Myc and anti-Smad3 antibodies. (F) Serum-starved HEK293 cells were transfected with WT Smad3, an Smad3 mutant construct containing only the MH2 domain (S229-425), BRCC3 plasmids, or an empty vector and then treated with TGF-β1 for 30 min. The level of K63-linked ubiquitination of Smad3 was determined by immunoprecipitation with an anti-Myc antibody, followed by immunoblotting with an anti-K63-Ub antibody. (G) Schematic diagram of BRCC3 interaction with the Smad3 MH2 domain. BRCC3 is depicted in brown and Smad3 in blue. Six β-sheets (green) and 3 loops (gray curve) of SMAD3-MH2 were observed to constitute the major binding interface with BRCC3. Given this, the Smad3 K333, K341, and K378 residues located at the binding interface were considered potential ubiquitination sites in the SMAD3-MH2 domain. (H) Serum-starved HEK293 cells were respectively transfected with 3 ng of 4 kinds of lysine residue mutations in Smad3 MH2 domain (i.e., K333, K341, K378, and K333/341/378) plasmids for 72 h and then treated with TGF-β1 (4 ng/ml) or the vehicle for 30 min. Cellular extracts were immunoprecipitated with anti-Myc. The immunoprecipitates were then subjected to immunoblotting analysis with anti-K63-Ub, anti-p-Smad3, and anti-Smad3. (I) Serum-starved HEK293 cells were co-transfected with 3 ng of WT-Smad3, or K333R plasmids together with AD-Ctrl or AD-*Brcc3* adenovirus for 72 h, and then treated with TGF-β1 (4 ng/ml) or the vehicle for 30 min. Cellular extracts were immunoprecipitated with anti-Myc. The immunoprecipitates were then subjected to immunoblotting with anti-Smad4 and anti-Myc. All inputs were determined in the same membrane by immunoblotting with α-tubulin. All experiments were repeated at least 3 times, and representative results are shown. (J) Serum-starved HEK293 cells were co-transfected with 3 ng of WT-Smad3, or K333R with AD-Ctrl or AD-*Brcc3* adenovirus for 72 h, and then treated with TGF-β1 (4 ng/ml) or the vehicle for 12 h. Total RNAs were extracted, and the expression of *Periostin* and *CTGF* messenger RNA (mRNA) was determined by real-time reverse transcription polymerase chain reaction (RT-PCR). Results are expressed as mean ± SEM. Statistical analysis: One-way ANOVA followed by Bonferroni multiple comparison tests.

Since we previously demonstrated that BRCC3 can bind to cytoplasmic Smad3 in response to cGMP treatment [23], we next tested whether the DUB activity of BRCC3 plays a role in antagonizing Smad3 signaling. Previous studies have shown that H122, H124, and D135, as critical amino acid residues, are essential for the deubiquitinating enzyme activity of BRCC3 [29]. To abrogate this catalytic function, we generated a catalytically inactive BRCC3 mutant (BRCC3-mut) by site-directed mutagenesis. In vitro functional assays revealed that relative to the WT BRCC3, the mutant BRCC3 exhibited a substantially diminished capacity to suppress Smad3 activation. Concurrently, the expression levels of extracellular matrix components—including CTGF, collagen I, and collagen III—were markedly elevated (Fig. S5A and B). These findings demonstrate that BRCC3’s inhibitory regulation of the TGF-β/Smad3 signaling pathway is strictly dependent on its deubiquitinating enzymatic activity. Of note, our co-IP analysis showed that TGF-β1 treatment significantly increased the K63-linked polyubiquitination of Smad3 in cells with AD-Ctrl, and BRCC3 knockdown by AD-*Brcc3* short hairpin RNA (shRNA) enhanced Smad3 K63-linked polyubiquitination, whereas infection of AD-*Brcc3* markedly decreased this TGF-β1-induced Smad3 polyubiquitination in CFs (Fig. 5A and B). These 2 complementary co-IP experiments with BRCC3 gain or loss of function suggested that BRCC3 reduces TGF-β1-induced K63-linked polyubiquitination of Smad3. By contrast, BRCC3 had no effects on the K48-linked polyubiquitination of Smad3 (Fig. S6A). To determine whether Smad3 ubiquitination is a prerequisite for its phosphorylation, we performed a time-course analysis of Smad3 ubiquitination and phosphorylation following TGF-β1 stimulation from 0 to 30 min. Ubiquitination of Smad3 was detectable as early as 5 min poststimulation, with the signal intensity progressively increasing over time; by contrast, Smad3 phosphorylation became detectable only at the 10-min time point and subsequently increased. These findings indicate that Smad3 ubiquitination precedes its phosphorylation (Fig. S6B). In addition, our in vivo study also showed that AMI increased the total myocardial K63-linked polyubiquitination level. However, cardiac-specific BRCC3 overexpression effectively decreased the whole myocardial K63-linked polyubiquitination level (Fig. S6C). Similarly, AMI increased the ubiquitination level of the Smad3 K63 site in myocardial tissue, while the overexpression of the cardiac-specific BRCC3 effectively reduced the ubiquitination level of the Smad3 K63 site (Fig. S6D).

To further define the BRCC3 binding motif associated with Smad3, we constructed Smad3 deletion constructs tagged with Myc (Fig. 5C). HEK293 cells were transfected with these constructs expressing truncated Smad3 of varying lengths. Co-IP experiments were then performed with anti-Myc and anti-BRCC3 antibodies. Endogenous BRCC3 could be co-immunoprecipitated with the full-length Myc-Smad3 (WT), S133-425, and S229-425, but not with S12-132 or S12-228 (Fig. 5D). These results were confirmed by reverse co-IP (Fig. 5E). Functionally, the K63-linked polyubiquitination of full-length Smad3 (WT) and S229-425 in TGF-β1-treated HEK293 cells was decreased by BRCC3 overexpression (Fig. 5F). These results suggest that the MH2 domain of Smad3 spanning amino acids 229 to 425 interacts with BRCC3.

To further investigate the mechanism by which BRCC3 affects SMAD3-MH2 ubiquitination, we performed protein–protein docking analysis to simulate the interactions between BRCC3 and SMAD3-MH2. According to the predicted binding mode, BRCC3 binds to SMAD3-MH2 mainly through electrostatic interactions. This was mainly because BRCC3 contains a stretch of negatively charged areas (L284 to E311), while SMAD3-MH2 has a positively charged groove (K378 to R386). Six β-sheets and 3 loops of SMAD3-MH2 were observed to constitute the major binding interface with BRCC3. Given this, K333, K341, and K378, located at the binding interface, were considered potential ubiquitination sites in the SMAD3-MH2 domain (Fig. 5G and Fig. S7). Next, we used the site-directed mutation strategy to construct different mutation site plasmids of Smad3. As expected, mutation of K333, but not K341 and K378, completely abolished TGF-β1-induced Smad3 K63-linked polyubiquitination. Of note, TGF-β1-induced Smad3 phosphorylation was significantly decreased in cells transfected with K333 or K333/341/378 (Fig. 5H). In addition, our co-IP analysis showed that in HEK293 cells transfected with WT Myc-Smad3 plasmid (WT-Smad3), TGF-β1 treatment markedly enhanced the interaction between WT Smad3 and endogenous Smad4 (i.e., Myc-Smad3 bound to Smad4), and BRCC3 overexpression effectively inhibited the binding of Myc-Smad3 to Smad4. By contrast, in HEK293 cells transfected with mutant K333-Smad3 (K333R), TGF-β1 treatment did not enhance mutant Myc-Smad3 bound to Smad4 compared with WT-Smad3. Furthermore, overexpression of BRCC3 did not affect the binding of mutant Myc-Smad3 to Smad4 (Fig. 5I). Functionally, transfection of the K333R plasmids not only attenuated TGF-β1-induced mRNA expression of *CTGF* and *Periostin* but also fully counteracted AD-*Brcc3*-mediated inhibitory action on extracellular matrix expression in HEK293 cells (Fig. 5J). These findings indicate that K63-linked ubiquitination at Lys333 of Smad3 serves as a critical marker for Smad3 activation, is essential for phosphorylation at Ser423 and Ser425, and facilitates interaction with Smad4. Moreover, BRCC3 deubiquitinates K63-linked polyubiquitin chains conjugated to Smad3 at Lys333, thereby suppressing Smad3 activation.

To further confirm that the K333 residue of Smad3 serves as the specific molecular target of BRCC3, we employed an adenovirus encoding BRCC3-targeting shRNA (Ad-*shBrcc3*) to achieve efficient BRCC3 knockdown in both cardiomyocytes and CFs. Subsequently, these cells were co-transfected with either WT Smad3 or the K333R mutant adenovirus, followed by exposure to hypoxia–glucose deprivation (OGD) or TGF-β stimulation—2 well-established paradigms for modeling myocardial injury. In cells reconstituted with WT Smad3, BRCC3 depletion markedly augmented Smad3 phosphorylation and significantly exacerbated pathological outcomes: elevated levels of myocardial injury biomarkers (creatine kinase–MB isoenzyme [CK-MB] and malondialdehyde), as well as increased extracellular matrix deposition mediated by fibroblasts (evidenced by up-regulated collagen I and collagen III expression) (Fig. S8A to D). In stark contrast, in cells expressing the Smad3-K333R mutant, BRCC3 knockdown failed to further enhance Smad3 activation; concomitantly, no significant increases were observed in CK-MB or malondialdehyde levels, nor in the expression of fibrosis-associated genes (collagen I and collagen III) (Fig. S8A to D). Collectively, these results provide direct biochemical and functional evidence for substrate specificity, demonstrating that BRCC3 exerts its regulatory effects on downstream signaling and pathophysiological outcomes exclusively through the deubiquitination of the K333 residue of Smad3.

To further validate the conclusion that BRCC3 exerts its protective effects specifically via modulation of the TGF-β/Smad3 pathway to suppress adverse cardiac remodeling, we conducted complementary experiments using the selective TGF-β1/Smad3 inhibitor IN-1 in *Brcc3*-KO mice subjected to MI. The results demonstrated that IN-1 treatment not only improved cardiac function—including reductions in LVESD and LVEDD and increases in LVEF and left ventricular fractional shortening (LVFS)—and attenuated myocardial fibrosis (as assessed by Masson’s trichrome staining) in WT mice post-MI but also partially ameliorated the exacerbated pathological phenotype observed in *Brcc3*-KO mice (Fig. S9A and B). Western blot analysis confirmed that IN-1 effectively suppressed MI-induced phosphorylation of Smad3 (p-Smad3) even in the absence of BRCC3 (Fig. S9C). Collectively, these findings establish a causal link between the adverse cardiac remodeling in *Brcc3*-KO mice and hyperactivation of the Smad3 signaling pathway. Thus, our study provides direct pharmacological evidence that BRCC3 mitigates post-MI cardiac remodeling predominantly through negative regulation of the TGF-β/Smad3 axis, and that pharmacological inhibition of this pathway with IN-1 can partially rescue the pathological phenotype associated with BRCC3 deficiency.

### BRCC3 alleviates ischemia-induced myocardial DNA damage

**Fig. 6.**
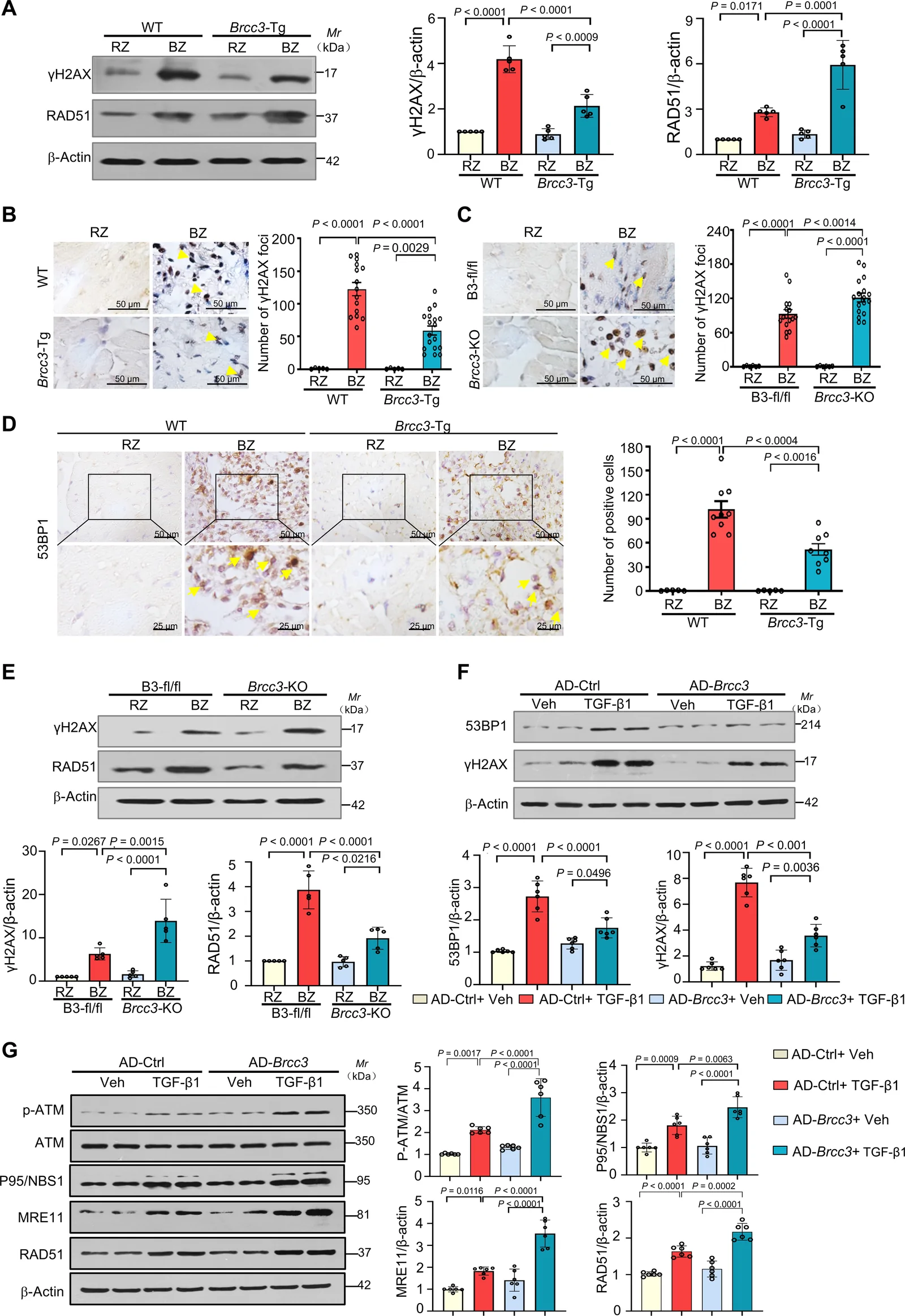
BRCA1/BRCA2-containing complex subunit 3 (BRCC3) overexpression reduces DNA double-strand break (DSB) and increases DNA damage repair. (A) *Brcc3*-Tg and wild-type (WT) littermate mice (*n* = 7 to 8 mice/group) underwent left anterior descending artery (LAD) ligation surgery. Tissues from the border zone area and the remote area (away from the infarct area) were extracted from the left ventricle. Western blot analysis of the protein levels of γH2AX and RAD51 with β-actin as the loading control. RZ, remote zone; BZ, border zone. (B) Immunohistochemical staining (200× magnification) of γH2AX foci in the border zone and remote area of the infarcted hearts of *Brcc3*-Tg and WT mice. Scale bar = 50 μm. (C) Immunohistochemical staining (200× magnification) revealed myocardial infarction (MI)-induced γH2AX expression in the border zone and remote area of the infarcted hearts in *Brcc3*-KO and age-matched littermate B3-fl/fl. Scale bar = 50 μm. (D) Immunohistochemical staining (200× magnification) of 53BP1 in left ventricles of *Brcc3*-Tg and WT mice. Scale bar = 50 μm (up) or 25 μm (down). (E) *Brcc3*-KO and age-matched littermate B3-fl/fl underwent MI surgery. Western blot analysis of γH2AX and RAD51 expression in the border zone area and the tissue away from the infarct area. β-Actin was the loading control. (F and G) Cultured cardiomyocytes were treated with 50 ng/ml transforming growth factor-β1 (TGF-β1) or the vehicle (Veh) for 48 h. Western blot analysis of DSB formation markers (i.e., γH2AX and 53BP1) and DNA damage repair response proteins such as phosphorylated ataxia-telangiectasia mutated (ATM) kinase and Mre11 complex including P95/NBS1, Mre11, and RAD51. β-Actin was the loading control. *n* = 7 to 11. Results are expressed as mean ± SEM. Statistical analysis: One-way ANOVA followed by Bonferroni multiple comparison tests.

It is well-known that sustained myocardial ischemia leads to the formation of large amounts of local reactive oxygen species and thus increases oxidative stress followed by cellular DNA damage, necrosis, and loss of cardiomyocytes in AMI. Because enhancement of DNA damage repair by preventing DNA DSB formation is the earliest confirmed biological function of BRCC3 [16], we tested whether BRCC3 potentiates protection of the heart against ischemia-induced DSB formation in MI mice. For this, we detected the phosphorylation of the histone variant H2AX (γH2AX) Ser139, a classical marker of DNA damage initiation and DSB induction [30]. The expression of γH2AX in the border zone of the infarcted heart in WT mice post-MI was markedly higher than that in the remote zone far from infarct zones. Importantly, ischemic injury increased DSB formation, and the DNA repair mechanism was activated in parallel, as revealed by an increase in RAD51 expression in the infarct border zone. Cardiac BRCC3 overexpression in *Brcc3*-Tg mice not only reduced the γH2AX level but also enhanced the RAD51 level compared with those in WT controls post-MI (Fig. 6A). Immunohistochemical staining showed that γH2AX foci were largely located in the infarct border zone rather than the infarct region in WT MI mice. The number of γH2AX-foci cells in the infarct border zone of *Brcc3*-Tg mice was significantly lower than that in WT mice (Fig. 6B), whereas the number of γH2AX foci in *Brcc3*-KO mice was higher than that in B3-fl/fl mice (Fig. 6C). As a control, expression of p53-binding protein 1 (53BP1), another marker of a DSB, was also lower in *Brcc3*-Tg than in WT mice in the infarct border zone (Fig. 6D). In agreement, cardiac-specific BRCC3 knockout in *Brcc3*-KO mice potentiated MI-induced γH2AX level but reduced the RAD51 level in the infarct border zone (Fig. 6E). These results from the in vivo study demonstrated that the protective effect of BRCC3 on cardiac function is at least to some extent related to the reduction of myocardial DNA damage in MI mice.

To establish a direct connection between the inhibition of TGF-β1–Smad3 signaling and DNA damage protection by BRCC3 in the heart, we further investigated whether sustained TGF-β1 challenge potentiates DNA damage response in neonatal mouse cardiomyocytes. Cardiomyocytes infected with AD-*Brcc3* or AD-Ctrl were challenged with 50 ng/ml TGF-β1 for 48 h. As shown in Fig. 6F, we found that excessive TGF-β1 stimulation not only led to increased expression of γH2AX and 53BP1 but also initiated DNA damage response repair mechanisms, characterized by an increase in ataxia-telangiectasia mutated (ATM, a DNA damage response key protein) kinase phosphorylation and up-regulation of the Mre11 complex including P95/NBS1, Mre11, and RAD51 [31]. Pretreatment with the specific Smad3 inhibitor (E)-SIS3 (10 μM) significantly suppressed TGF-β1-induced up-regulation of γH2AX and 53BP1, suggesting an Smad3-dependent mechanism of TGF-β1 on DNA damage response (Fig. S9A). Similarly, overexpression of BRCC3 effectively decreased the expression of γH2AX and 53BP1, while it augmented the protective mechanism against DNA damage by up-regulating ATM phosphorylation and expression of P95/Nbs1, Mre11, and RAD51 in cardiomyocytes treated with a high dose of TGF-β1 treatment (Fig. 6G). Moreover, inhibition of Smad3 by (E)-SIS3 could fully abolish the effect of BRCC3 on TGF-β1-induced DSB formation, suggesting that Smad3 is required for BRCC3 in antagonizing TGF-β1-induced DSB formation (Fig. S9A).

Lastly, because sustained myocardial ischemia-induced oxidative stress directly leads to cellular DNA damage, we next tested whether BRCC3 confers cardiomyocyte protection against oxidative stress caused by H_2_O_2_. As shown in Fig. S9B, H_2_O_2_ treatment significantly increased γH2AX and RAD51 foci in cardiomyocytes infected with AD-Ctrl. However, infection of cardiomyocytes with AD-*Brcc3* effectively decreased γH2AX expression and foci formation induced by H_2_O_2_ while increasing RAD51 expression and foci formation (Fig. S9C). Collectively, these results from in vivo and in vitro studies suggest that both excessive activation of TGF-β1–Smad3 signaling and oxidative stress due to sustained ischemia trigger myocardial DNA damage response and DSB formation, and BRCC3 overexpression alleviates DSB formation and triggers the ATM-mediated DNA damage repair in cardiomyocytes.

## Discussion

The current study is the first to show that BRCC3, through its negative regulation of TGF-β1–Smad3 signaling, confers protection to the mouse heart against acute ischemia-induced DNA damage, cardiac remodeling, and dysfunction. Using mouse models, we demonstrated that AMI reduced BRCC3 expression in the stressed mouse heart. As a specific deubiquitinating enzyme targeting K63-linked polyubiquitin chains, BRCC3 efficiently removes K63-linked polyubiquitination at lysine 333 of Smad3, thereby inhibiting Smad3 activation—a nonproteolytic, signal-transducing posttranslational modification. In line with this newly deduced mechanism, cardiac-specific BRCC3 overexpression in mice markedly reduced infarct size, ventricular dilation, cardiac fibrosis, and myocardial DNA DSB formation and improved survival after AMI. In contrast, myocardial ablation of BRCC3 greatly exacerbated acute ischemia-induced myocardial damage, cardiac remodeling, and dysfunction, which contributed to DNA DSB formation and death. In line with our findings, previous studies reported that macrophage-specific TREM2 deficiency exacerbates cardiac dysfunction and enlarges the infarct area in a mouse sustained LAD-ligated AMI model by up-regulating *SLC25A53* transcription [32]. Gu et al. [33] found that cardiac-specific overexpression of NLR family CARD domain containing 5 (*NLRC5*) reduces the MI area and improves cardiac function by enhancing autophagic flux of the CAVIN1/CAV1 axis in the same AMI model. Overall, these results reveal a critical role for BRCC3 in mitigating cardiac remodeling and myocardial DNA damage while augmenting DNA DSB repair by inhibiting TGF-β1–Smad3 signaling in cardiomyocytes and CFs.

To infer the translational relevance of BRCC3 involvement in cardiac disease, we also tested the expression levels of BRCC3 in clinical samples. We found that the serum level of BRCC3 is detectable in healthy control subjects. Although we presently do not know exactly where it comes from or how, the results imply that BRCC3 is likely to exist in blood plasma in a naturally secreted manner. Interestingly, we showed that down-regulation of myocardial BRCC3 expression and serum BRCC3 levels in patients with ischemic heart disease were associated with the development of cardiac dysfunction. However, it is noted that a definitive causal relationship between BRCC3 and prognosis in patients suffering from ischemic heart disease needs further investigation involving longer durations and larger sample sizes.

We previously reported that although disruption of TGF-β1–Smad signaling by overexpression of dominant negative type II TGF-β receptor inhibited transverse aortic constriction-induced cardiac fibrosis and remodeling [34], mice showed ventricular dilation and cardiac dysfunction at 120 d after surgery, so directly blocking TGF-β1–Smad signals at the receptor level might not be optimal to mitigate cardiac remodeling. Although TGF-β1 signal cascade-related Smads, including Smad2, Smad3, Smad4, and inhibitory Smad7, are involved in pathological cardiac remodeling [35–37], their functional differences in cardiac remodeling show distinct cell type and stimulus context dependence [4,6]. Previous studies have shown that cardiac tissue from patients with heart failure with reduced ejection fraction or heart failure with preserved ejection fraction and AMI exhibit increased levels of TGF-β1 and activated Smad3 [38]. Smurf2 has been shown to promote the degradation of Smad3 by increasing Smad3 K48-linked polyubiquitination [9]. Therefore, Smad3 expression remains unchanged likely due to the increase of Smurf2 24 h after AMI, which is different from the up-regulation of the entire TGF-β signaling pathway in the chronic phase of MI [39]. By contrast, the phosphorylation level of Smad3 was substantially increased. In a transgenic mouse model, we showed that cardiac-specific overexpression of BRCC3 leads to inhibition of TGF-β1–Smad3 activation after AMI (Fig. 3A), suggesting the existence of an intrinsic association between TGF-β1–Smad3 and BRCC3. However, BRCC3 does not appear to affect TGF-β1-induced Smad2 activation (Fig. S5B), suggesting that Smad3, but not Smad2, functions as a key target of BRCC3 in the heart.

Accumulating evidence has demonstrated that posttranslational modifications of Smad3, such as phosphorylation, acetylation, methylation, and K48-linked ubiquitination, are involved in its activation, translocation, and stability [8–10]. Classically, phosphorylation of Ser423/425 at its C-terminus is a hallmark of Smad3 activation in response to TGF-β1 stimulation [8]. However, Smad3 undergoes Nedd4L-mediated K48-linked polyubiquitination, thus leading to Smad3 degradation that attenuates TGF-β signaling [40]. Other Lys residues of Smad3 can be mono-ubiquitinated by the E3 ligase Smurf2 [9]. Although mono-ubiquitination of Smad3 does not appear to affect its phosphorylation or stability, this mode of posttranslational modification inhibits Smad3 from transactivating its target genes [9]. By contrast, USP15 can activate Smad3-mediated transactivation by deubiquitinating K48-linked mono-ubiquitinations [41]. Here, we provide new evidence supporting that BRCC3-catalyzed K63-specific deubiquitination modification functions as a novel regulatory mechanism for Smad3 activation involved in TGF-β1 signaling and cardiac remodeling. That is, TGF-β1 treatment of adult mouse CFs increased Smad3 Ser423/425 phosphorylation as well as K63-linked ubiquitination. Overexpression of exogenous BRCC3, while effectively deubiquitinating Smad3, inhibited TGF-β1-induced Smad3 phosphorylation, nuclear translocation, and extracellular matrix gene expression. However, when the BRCC3 enzyme loses its ubiquitin-degrading activity (due to a mutation at the active site), overexpression of BRCC3 is unable to inhibit the activation of Smad3. Further, we showed that mutation of Smad3 at K333 not only effectively abolished TGF-β1-induced Smad3 K63-linked polyubiquitination but also attenuated its Ser423/425 phosphorylation. More importantly, we showed that inhibition of Smad3 by the specific inhibitor (E)-SIS3 effectively covered the inhibitory action of BRCC3 on TGF-β1-induced *CTGF* and *Periostin* mRNA expression. Mechanistically, the mutation of K333 of Smad3 fully abolished the inhibitory effect of BRCC3 on TGF-β1-induced Smad3 binding to Smad4. These results suggest that both K63-linked polyubiquitination at K333 and phosphorylation at Ser423/425 may be indispensable for the full activation and function of Smad3 in the heart. Ovarian tumor deubiquitinase-1 (OTUB1) is a cysteine protease that hydrolyzes the isopeptide bonds between ubiquitin and its target or other ubiquitin molecules [42]. It has been shown to interact with phosphorylated SMAD2/3 (p-Smad2/3) and prevent Smad2/3 polyubiquitylation formation by binding to and inhibiting the E2 ubiquitin-conjugating enzyme UBE2N (also known as UBC13), independent of its catalytic activity [43,44]. Recently, OTUB1-mediated reduction of K63-linked and K48-linked polyubiquitination of Smad3 together conferred the transcription of several genes involved in vascular endothelial-to-mesenchymal transition and remodeling in endothelial cells [45], suggesting that K63-linked deubiquitination of Smad3 might be regulated in a cell-type-specific and context-dependent manner. Our previous co-IP followed by LC–MS/MS analysis addressed an interaction between BRCC3 and Smad3 [23]. Given that there is no evidence to support the existence of enzyme-activity-independent deubiquitination effect of BRCC3 so far, the results indicate that BRCC3 might act as a direct DUB for Smad3 to remove its K63-linked polyubiquitination.

Functionally, our gain- and loss-of-function studies of BRCC3 in cardiomyocytes showed that increased myocardial expression of BRCC3 in vivo was associated with alleviation of K63-linked polyubiquitination and pathological cardiac remodeling and improvement of cardiac dysfunction after AMI. After MI, Smad3 phosphorylation in the left ventricle was attenuated in *Brcc3*-Tg mice compared with that in WT controls. This attenuation of Smad3 activity in *Brcc3*-Tg mice was consistent with reduced collagen accumulation and extracellular matrix deposition. Cardiomyocytes account for approximately 80% of the heart’s volume and are the main cells that make up the heart. Similar to previous studies [46,47], BRCC3 is almost not expressed in the myocardial tissue of mice with cardiomyocyte-specific *Brcc3* knockout. Myocardial *Brcc3* knockdown exacerbated MI-induced cardiac hypertrophy and fibrosis. Although the main functional cells of the fibrosis are CFs, our co-culture results provide good support for these 2 cell–cell interactions as well as these in vivo findings, which BRCC3 overexpression in cultured cardiomyocytes effectively inhibited TGF-β1-induced Smad3 activation in co-cultured CFs by an unknown BRCC3-driving paracrine mechanism. As a functional K63-linked DUB, it is now clear to us that Smad3 should not be the only target of BRCC3 in the heart. Meanwhile, the functional comparative studies highlight the possibility that the regulation of myocardial K63-linked polyubiquitination by BRCC3 may be an alternative strategy to prevent cardiac remodeling.

Basically, BRCC3 acts as the catalytic subunit for 2 multiprotein complexes: BRCA1-A and BRCC3 isopeptidase complex (BRISC). In the BRCA1-A complex, ABRAXAS interacts with the DNA repair protein RAP80 to provide a high-affinity binding site that sequesters BRCA1 at DSB sites in a γH2AX- and MDC1-dependent manner [48]. BRCC3, via its DUB activity, promotes BRCA1-A complex accumulation at DSB sites and enhances DSB repair by limiting DNA end resection [16]. A previous study showed that ischemic induction of BRCA1 in human adult and fetal cardiac tissues contributes to protecting cardiomyocytes against DNA damage and apoptosis as well as maintaining cardiac function during ischemic or genotoxic stress [49]. Loss of BRCA2 in cardiomyocytes could markedly increase apoptosis, thus resulting in heart failure and death [50]. In the BRISC complex, BRCC3, combined with SHMT2 and KIAA0157, is implicated in regulating interferon signaling and viral protein degradation [51]. As a DNA damage sensor, the Mre11 complex, composed of Mre11, Rad50, and P95/NBS1, is required for ATM kinase activation and orchestrates all stages of the DNA damage response, including sensing the initial lesion, activating checkpoint signaling, driving specific repair pathways, and structurally bridging the participating DNA molecules together, and thus plays an essential role in promoting DNA nonhomologous end-joining repair and homology-directed repair [52]. Interestingly, Smad3 has been shown to form a complex with BRCA1 in the nucleus and suppress BRCA1-dependent DSB repair in human breast epithelial cells in response to a DNA-damaging agent [53]. Consistent with this, here we demonstrated that BRCC3 overexpression can increase the expression of the Mre11 complex and ATM phosphorylation in cells in response to sustained TGF-β1 and H_2_O_2_ challenge (mimicking a state of excessive TGF-β1 activation and oxidative stress in AMI), suggesting a beneficial role of BRCC3 in promoting DNA damage repair response. In cells with low proliferative potential, DNA damage and DSB repair occur in stressed cardiomyocytes [54,55]. In our study, AMI resulted in myocardial BRCC3 down-regulation, accompanied by an increase in DSB formation, as confirmed by the induction of γH2AX and RAD51. These results indicate that DSB repair was hindered under acute cardiac stress, which was due at least in part to BRCC3 down-regulation. This thesis is supported by cardiac-specific *Brcc3* overexpression reducing γH2AX levels but increasing RAD51 levels after AMI. Conversely, *Brcc3* knockdown promoted DSB and inhibited RAD51 levels. Hence, elevated BRCC3 levels would contribute to decreased DSB formation while enhancing DNA damage repair. Consistent with our study, Shukla et al. [49] demonstrated that BRCA1 deficiency in cardiomyocytes impaired DSB repair, activated p53-mediated pro-apoptotic signaling, and increased cardiomyocyte apoptosis, thus increasing mortality in response to ischemic or genotoxic stress. Our data together with those from Shukla et al. demonstrate that BRCC3 is involved in cardiac remodeling at least in part by alleviating DNA damage under acute ischemic stress conditions.

In summary, our findings provide new insights into the prevention of cardiac remodeling and dysfunction under ischemic conditions through targeted modulation of Smad3 K63-linked polyubiquitination by BRCC3 to suppress ischemia-induced excessive activation of TGF-β1–Smad3 signaling and subsequent myocardial DNA damage, which may represent an alternative approach and therapeutic strategy for AMI via pharmacological intervention involving BRCC3. The next step for researchers involves employing molecular simulation docking technology to identify BRCC3-specific agonists, as well as utilizing protein purification techniques to isolate the BRCC3 protein. These efforts aim to facilitate the clinical application of BRCC3 in the treatment of AMI.

## Materials and Methods

### Generation of cardiac-specific *Brcc3*-overexpressing transgenic mice

Transgenic mice expressing the mouse *Brcc3* gene in cardiac muscle were created by using the cardiac-specific promoter α-myosin heavy chain (*α-MHC*), as we previously described [56]. Briefly, a 5.4-kb fragment of the murine *α-MHC* gene promoter and 876-kb mouse *Brcc3* complementary DNA (cDNA) were subcloned into the PRP.EX3D plasmid to obtain the expression clone PRP.EX3D-*αMHC*-*Brcc3*-IRES/EGFP by gateway technology. The plasmid was digested with NotI to generate a 10,816-bp DNA fragment consisting of the *α-MHC* gene promoter, *Brcc3* cDNA, and IRES/EGFP. The construct was microinjected into the pronuclei of single-cell fertilized C57BL/6J embryos to generate *Brcc3*-Tg transgenic mice. Polymerase chain reaction (PCR) analysis with genomic DNA isolated from tail tips was used to identify offspring carrying the eGFP transgene with the specific primer: forward, ACG TAA ACG GCC ACA AGT TC; reverse, GAT CTT GAA GTT CAC CTT GAT GC. Hearts from 8- to 12-week-old *Brcc3*-Tg mice were collected to measure the mRNA and protein levels of myocardial BRCC3. Age-matched littermates of male WT mice were used as controls.

### Generation of cardiac-specific *Brcc3* gene conditional knockout mice

*Brcc3*^flox/flox^ mice were generated by Cyagen Bioscience (Guangzhou, China). Briefly (Fig. S1A), LoxP sites were cloned into the *Brcc3* gene (GenBank accession no. NM_001166457.1; Ensembl ENSMUSG00000031201) via Flp recombination flanking exon 5. To engineer the targeting vector, homology arms and conditional knockout (CKO) regions were generated by PCR with the BAC clone RP23-360P8 from the C57BL/6J library. In the targeting vector, the Neo cassette was flanked by Frt sites, and the CKO region was flanked by LoxP sites. After Flp-mediated recombination, the CKO allele was obtained. To generate *Brcc3*^flox/flox^ mice, the Neo-deleted heterozygote mice (*Brcc3*^flox+/−^) were crossed with *Brcc3*^flox+/−^ mice. The offspring homozygote mice were confirmed by PCR genotyping with specific primers for m*Brcc3* forward, TGA GGG AGT TAT CAA GTT TTG GGG, and m*Brcc3* reverse, TTT CAG TTC CAG GCC AGC TCT. Ablation of the *Brcc3* gene was achieved by crossbreeding between *Brcc3*^flox/flox^ and *α-MHC-Cre*^+/−^ mice to generate *Brcc3*^flox/flox^ × *α-MHC-Cre*^+/−^ mice. Cre-mediated excision of exon 5 resulted in a frameshift mutation from exons 4 to 6, leading to an early translational stop and subsequent *Brcc3* knockout. *Brcc3*^flox/flox^ × *α-MHC-Cre*^+/−^ mice were then crossed with *Brcc3*^flox/flox^ mice to obtain conditional *Brcc3*^−/−^ mice (*Brcc3*-KO) and WT littermate controls (*Brcc3*^flox+^/Cre^−^) confirmed by PCR genotyping with the following primers: Cre forward, 5′-GAA CGC ACT GAT TTC GAC CA-3′; Cre reverse, 5′-GCT AAC CAG CGT TTT CGT TC-3′; m*Brcc3* forward, 5′-ATT GAA CCC AGG TCC ACT GCA AG-3′; and m*Brcc3* reverse, 5′-TTT CAG TTC CAG GCC AGC TCT-3′ (Fig. S1B). BRCC3 deletion in the heart was verified by Western blot analysis with anti-BRCC3 antibody (Fig. S1C).

### RNA-seq and in silico analysis

To investigate the differentially expressed genes potentially related to BRCC3 during MI, we analyzed a GEO RNA-seq dataset (GSE48060) containing data for patients with MI with any recurrent events over an 18-month follow-up. The heatmap was generated using the online tool Morpheus (https://software.broadinstitute.org/morpheus). We analyzed the GEO RNA-seq dataset (GSE153485) to compare the expression of BRCC3-related differential genes in myocardial tissue 24 h after MI in mice. One extremum was discarded in the MI group and the sham group.

To compare the levels of *Brcc3* in different cell types in mouse heart, single-nucleus RNA-seq data from GEO with accession number GSE214611 were downloaded and analyzed using the “Seurat” package (version 5.3.1). A total of 56,002 nuclei from mouse heart tissues (3 normal mice and 5 mice underwent LAD ligation for 24 h) were included for analysis after a series of standard quality procedures using the “NormalizeData”, “FindVariableFeatures”, “ScaleData”, “RunPCA”, “FindNeighbors”, “FindClusters”, and “RunUMAP” functions. Cells were clustered based on marker genes. After extracting the normalized *Brcc3* gene levels using the Fetch function, cells that did not express this gene were filtered out; the expression levels of *Brcc3* were compared between different clusters and cell types.

For RNA-seq and data analysis, neonatal rat cardiomyocytes were transfected with AD-*Brcc3* or AD-Null (100 multiplicity of infection) for 72 h, and total RNA was extracted with TRIzol. The RNA quality was measured with NanoDrop One Bioanalyzer and high-quality RNA (RNA integrity number ≥8.0) was used. RNA libraries were prepared by using the VAHTS Universal V8 RNA-seq Library Pre Kit (Illumina) following the manufacturer’s protocols. The clustering of the index-code samples was performed on a cBot Cluster Generation System with TruSeq PE Cluster Kit v3-cBot-HS (Illumina) and sequenced on an Illumina NovaSeq 6000 platform. The low-quality filtered sequences were then filtered for ribosomal RNA using the sortmerna v4.3.4 tool. The filtered sequence data were then compared to the reference genome (*Rattus norvegicus* mRatBN7.2) using the STAR v2.7.10a tool. Then, quality assessment was conducted using RSeQC v4.0.0, QualiMap v2.2.2, featureCounts v2.0.1, and Preseq v3.1.1. The results of RNA comparison were quantitatively analyzed using the Salmon v1.9.0 tool. The transcript and its annotations refer to the ENSEMBL 110 database. The quantitative results of mRNA expression were obtained by quantitative analysis. R-packet DESeq2 was used to screen gene counts obtained from RNA-seq for differentially expressed genes between AD-Null and AD-*Brcc3* groups (log_2_ fold change >1 or <−1; false discovery rate <0.1). Enrichment analysis of differentially expressed genes was performed using the online tool DAVID (https://davidbioinformatics.nih.gov). The program was run with default settings, with significant Gene Ontology terms and pathways identified ranking by −log_10_(false discovery rate) (obtained by hypergeometric test) shown in Fig. 1C.

To analyze the BRCC3/Smad3 protein–protein interaction, Smad3–BRCC3 docking was performed by using ZDOCK 3.0.2 on the ZDOCK server (http://zdock.umassmed.edu). The precise 3-dimensional protein structures of BRCC3 (Protein Data Bank [PDB] ID: 6R8F) and SMAD3 (PDB ID: 5XOC) were obtained from the PDB. Ten possible interaction solutions were generated. The docked protein–protein complex was selected based on the maximum number of intermolecular ionic and hydrogen bonds established between BRCC3 and SMAD3. According to the best predicted binding mode, BRCC3 could bind to the SMAD3-MH2 domain very well.

### Isolation and culture of adult mouse CFs and neonatal mouse cardiomyocytes

CFs were isolated and cultured from C57BL/6 mice as described previously [57]. Briefly, C57BL/6J mice were euthanized by cervical dislocation to preserve the physiological properties of the cardiac cells. We did not use any anesthetic agent. Hearts were excised, minced, and digested with collagenase type 4 (100 U/ml) and trypsin (0.6 mg/ml) at 37 °C for 30 min. The digestion was repeated 5 to 6 times. The cell supernatant was centrifuged at 180×g for 10 min and resuspended in Dulbecco’s modified Eagle medium (DMEM) with 15% fetal bovine serum. Cells were plated into laminin-coated 100-mm dishes (BD BioCoat, BD Biosciences) and allowed to attach for 45 min before the first medium change, which removed weakly adherent myocytes and endothelial cells. Cultures were assessed for admixture of endothelial cells, macrophages, and myofibroblasts by immunofluorescence staining for CD31, F4/80, and α-smooth muscle actin, respectively. More than 95% of cultured cells were negative for these markers and had the typical appearance of fibroblasts. Passage 1 CFs were used for the experiments. As previously stated [58], C57BL/6J mice aged 1 to 3 d were euthanized by cervical dislocation to preserve the physiological properties of the cardiac cells and facilitate the extraction of cardiomyocytes. We did not use any anesthetic agent. For neonatal cardiomyocyte isolation, ventricles were digested with 0.1% trypsin and 80 U/ml collagenase II and centrifuged. Isolated cells were pre-plated for 90 min to remove noncardiac cells. Cardiomyocytes were resuspended in DMEM containing 100 mg/ml streptomycin and penicillin and 10% fetal bovine serum and seeded into 35-mm dishes. To inhibit noncardiomyocyte growth, 100 μmol/l 5-bromo-2′-deoxyuridine was added. After incubation at 37 °C in humidified air with 5% (v/v) CO_2_ for 24 h, cardiomyocytes were infected with adenovirus vector for 3 d and then deprived of serum and incubated for another 24 h before treatment.

### Acute myocardial infarction

AMI was induced by permanent coronary artery ligation, as described [59]. Adult (8 to 10 weeks old) male C57BL/6J mice were anesthetized with ketamine (80 mg/kg, intraperitoneal) and xylazine (5 mg/kg, intraperitoneal), fixed in the supine position on a sterile operating table, and shaved, and the left chest area was disinfected. Mouse incisors were fixed to facilitate endotracheal intubation, and the mouse was connected to a small animal ventilator. The mouse model of AMI was created by ligating the LAD. A 1-cm incision was made along the axilla to the xiphoid process of the sternum. The pectoralis major and pectoralis minor were isolated by blunt separation, and the chest cavity was exposed from the third intercostal space. The intercostal space was opened with a retractor to fully expose the heart, and the pericardium was carefully removed. The LAD was ligated with an 8-0 silk suture under the left auricle. The success of the procedure was assessed by observing the pallor of the anterior wall of the left ventricle and the elevation of ST segments on the electrocardiogram. The thorax was closed, and the skin was sutured. After the mouse resumed spontaneous breathing, the ventilator was removed. The same procedure was performed on the sham operation group except for LAD ligation. All animal experiments and protocols were approved by the Affiliated Hospital of Yangzhou University Ethics Committee (No. 2017012-2017-02) and performed in accordance with the guidelines from Directive 2010/63/EU of the European Parliament. The mice were humanely euthanized by inhalation of carbon dioxide, after which the heart tissue was harvested. Animal care and surgical procedures adhered to the principles of animal care outlined by the National Society for Medical Research and the Guide for the Care and Use of Laboratory Animals (National Institutes of Health [NIH] publication).

### Echocardiography studies

Transthoracic echocardiography was performed using a Vevo 3100 machine with an MX550S transducer (Visual Sonics, Toronto, Canada) under isoflurane anesthesia (1% to 1.5% at a flow rate of 1 l/min oxygen). LVEDD (mm) and LVESD were measured to assess left ventricular systolic function. LVFS (%) and LVEF (%) were calculated as follows: FS = [(LVEDD − LVESD)/LVEDD] × 100% and EF = [(LVEDD^3^ − LVESD^3^)/LVEDD^3^] × 100%. All data were acquired and analyzed by a single echocardiographer blinded to the groups.

### Immunohistochemical analysis

As previously described, left ventricular tissue samples were fixed, embedded in paraffin, and sectioned into 5-μm slices for staining [60]. Antigen retrieval was performed by boiling, and then sections were placed in 3% H_2_O_2_ at room temperature for 5 to 10 min to eliminate endogenous peroxidase activity. After washing with phosphate-buffered saline (PBS), 10% normal goat serum was incubated at room temperature for 1 h. The primary antibody was added, and the mixture was incubated at 4 °C overnight. The primary antibodies used in the study were anti-collagen I (ab6308), anti-collagen III (ab7778), anti-γH2AX (ab2893), and anti-53BP1 (ab175933, Abcam). For immunohistochemical staining, the slices were washed 3 times in PBS and incubated with a peroxidase-conjugated secondary antibody for 1 h. The expression of the target protein was measured using a DAB kit (Vector Biolabs, USA). For immunofluorescence, slices were incubated with a fluorescence isothiocyanate-conjugated secondary antibody. To examine nuclei, we treated each tissue section with 4′,6-diamidino-2-phenylindole (DAPI; Cat. D4592, Sigma-Aldrich). The sections were examined under a Nikon Ti-U inverted epifluorescence microscope equipped with appropriate filters (Nikon) at 100× magnification. Five randomly selected fields from each slide were measured by an observer blinded to the groups, and mean values were used for statistical analysis.

### Masson’s trichrome staining

Paraffin-embedded tissue sections were used for Masson’s trichrome staining with an SABC kit (Wuhan Boster Biological Technology, Wuhan, China). Ten randomly selected fields per section were used to calculate the proportion of positive staining to reflect the degree of cardiac fibrosis by light microscopy with color threshold analysis using NIH ImageJ.

### TTC staining

The area of the MI was measured by 2,3,5-triphenyl tetrazolium chloride (TTC) staining as described previously [61]. Freshly harvested heart tissue was cut into 2-mm-thick slices from the ligation site, placed in 1% TTC solution, incubated at 37 °C for 15 min, and then fixed overnight with 4% formaldehyde. Pictures were taken with a digital camera and analyzed using ImageJ.

### Adenoviral vector generation

The mouse *Brcc3* gene was amplified by PCR with pDEST-LTR-N-Flag-HA-*Brcc3* (Cat. 22540, Addgene, Cambridge, MA) as a template and then cloned into the adenovirus shuttle vector pDown-MCS-IRES/EGFP to create the recombinant plasmid pDown-*Brcc3*-GST-IRES-EGFP. The expression cassette of *Brcc3*-GST-IRES-EGFP was transferred to pAV.Desld with LR recombinant reaction to create the expression plasmid pAV.Ex1d-CMV-*Brcc3*-GST-IRES-EGFP, by Cyagen Bioscience (Guangzhou, China). The positive clone was linearized and transfected into 293A cells for packaging, and the amplified adenovirus *Brcc3* (AD-*Brcc3*) was identified. An adenovirus containing an empty vector with an eGFP tag was the AD-Ctrl control. To ablate the expression of endogenous BRCC3, the specific short hairpin RNA (shRNA) targeting the mouse *Brcc3* gene (AD-GFP-U6-m*Brcc3*-shRNA, Cat. shADV-254071) and scramble control shRNA (AD-GFP-U6-shRNA, Cat. 1122) were purchased from Vector Biolabs (Malvern, PA). Large-scale amounts of recombinant adenovirus were amplified in HEK293 cells and purified. Viral titers were determined by plaque assay on the HEK293 cell monolayer. Experiments were conducted at a virus amount of 100 multiplicity of infection. After 4 h of incubation, the medium was removed, and cells were incubated in an appropriate medium for 72 h. Transfection efficiency was assessed under a fluorescence microscope.

### Plasmid construction and transfection

Expression constructs encoding the various human Smad3 domain-deletion mutant genes were prepared by PCR and cloned into the pRP[Exp]-eGFP-CAG mammalian expression vector. Their sequences were confirmed by DNA sequencing. For overexpression of WT and mutant Smad3 in HEK293 cells, cells were grown in 100-mm dishes to ~70% confluence, and DNA transfection was performed by lipofection. After 72 h of transfection, serum-starved cells were treated with the vehicle or TGF-β1 (4 ng/ml) for 1 h and harvested. Whole-cell lysates were used for co-IP analysis with an antibody against Myc or Smad3.

### Co-IP and Western blot analysis

Total protein from heart tissues was extracted using radioimmunoprecipitation assay lysis buffer (Beyotime, Beijing). Cells were lysed in co-IP buffer (120 mM NaCl, 20 mM Tris, pH 7.5, 2 mM EDTA, 1% Triton X-100, 1 mM sodium vanadate, 10% glycerol) containing 0.5 mM phenylmethylsulfonyl fluoride and 20 mg/ml aprotinin and then centrifuged at 12,000×g for 15 min at 4 °C. Protein concentration was determined using the Bio-Rad protein assay (Bio-Rad, Richmond, CA). Following cell lysis, 600 μg of total protein per sample was precleaned with normal rat/mouse immunoglobulin G (IgG) and protein A/G-plus beads (sc-2003, Santa Cruz Biotechnology) and then immunoprecipitated with antibodies against anti-Smad3 (sc-8332, Santa Cruz Biotechnology), anti-BRCC3 (ab108411), anti-Ubk63 (ab179434, Abcam), and anti-Myc (M192-3, MBL, Japan) at 4 °C overnight. The bound proteins on protein A/G-plus beads were washed with co-IP buffer, centrifuged, eluted with 2× sample loading buffer, boiled at 95 °C for 5 min, and stored at −80 °C until use. Equal amounts of proteins were loaded onto 7.5% or 12% sodium dodecyl sulfate–polyacrylamide gel electrophoresis gels and transferred to polyvinylidene fluoride membranes (Millipore, Billerica, MA). After blocking with 5% (w/v) skim milk in Tris-buffered saline containing 0.1% (v/v) Tween 20 (TBST) for 1 h, membranes were incubated with various primary antibodies diluted in 5% (w/v) skim milk in TBST at 4 °C overnight. The primary antibodies used in this study were anti-p-Smad3 (#9520), anti-Smad3 (#9523), anti-BRCC3 (ab108411), anti-Smad2, anti-Ubk63 (ab179434), anti-γH2AX (ab2893), and anti-Rad51 (ab133534, Abcam). Anti-α-tubulin (sc-8035), anti-β-actin (sc-47778), anti-Myc, and anti-glyceraldehyde-3-phosphate dehydrogenase (anti-GAPDH; sc-365062, Santa Cruz Biotechnology) were used as loading controls. The membranes were washed 3 times in TBST buffer and incubated with horseradish peroxidase-conjugated secondary antibody for 2 h. Bands were visualized using the Pierce ECL Plus Western Blotting Substrate Kit (Thermo, USA) and quantified using NIH ImageJ. Each co-IP experiment was repeated at least 3 times.

### Real-time RT-PCR analysis

The levels of mRNA were determined by real-time reverse transcription PCR (RT-PCR). The total RNA of heart tissue and cultured cells was isolated by using TRIzol reagent (Invitrogen, USA). Equal amounts of RNA (1 μg) were reverse-transcribed by using the Reverse Aid First Strand cDNA Synthesis Kit (Thermo, USA). Real-time RT-PCR was performed using SYBR Green reagent (Cat. 4367659, Applied Biosystems) on an ABI PRISM 7500 sequence detection system (Applied Biosystems, CA). The specific primers were as follows: *Brcc3*: forward, 5′-TTG GAG CAA AAC CAG CAG CAT-3′; reverse, 5′-TCG CCA TTT ATC ACC TTC ATC G-3′; *CTGF*: forward, 5′-CGG TCC TTG GGC TCG TCA-3′; reverse, 5′-GCA CTT GCC TGG ATG GGG-3′; *Periostin*: forward, 5′-TGA GCT ACT GAA TGC CTT AC-3′; reverse, 5′-CAA TGA CAT GGA CGA CAC-3′; *GADPH*: forward, 5′-GTT GTC TCC TGC GAC TTC A-3′; reverse, 5′-GTG GTC CAG GGT TTC TTA CT-3′; *ANP*: forward, 5′-GCT TCC AGG CCA TAT TGG AG-3′; reverse, 5′-GGG GGC ATG ACC TCA TCT T-3′; and *β-MHC*: forward, 5′-ACT GTC AAC ACT AAG AGG GTC A-3′; reverse, 5′-TTG GAT GAT TTG ATC TTC CAG GG-3′. The cycle threshold (Ct) values of the target mRNA were normalized to *GADPH* levels as the internal control. Samples were compared by the relative (comparative) Ct method. Fold induction or repression was measured relative to controls and calculated after adjusting for GAPDH RNA (endogenous control).

### Immunofluorescence analysis

Treated cells were washed with PBS, fixed with 4% formaldehyde, and permeabilized with 0.5% Triton X-100. The fixed cells were blocked with 10% normal goat serum and then incubated with anti-Smad3 or normal rabbit/mouse IgG antibody at 4 °C overnight. Slides were incubated with a Texas Red-conjugated anti-rabbit secondary antibody (1:100, Cat. No. TI-1000, Vector Biolabs) and a fluorescein-conjugated anti-mouse secondary antibody (1:100, Cat. No. FI-2000, Vector Biolabs) for 1 h at room temperature. Nuclei were stained with DAPI (50 ng/ml) in PBS for 15 min. Coverslips were washed, mounted with 90% glycerol, and visualized by confocal laser microscopy (400×). Images were randomly acquired from different fields.

### Cell co-culture

Neonatal mouse cardiomyocytes were seeded onto the surface of the insert membrane with a 0.4-μm pore size in the upper layer of a 6-well plate. The lower part of the 6-well plate was seeded with neonatal CFs. After 48 h of culture, the complete medium was replaced and cardiomyocytes were infected with AD-*Brcc3* or AD-Ctrl control virus. At 48 h after virus infection, the medium was aspirated and washed with PBS. Cultured cardiomyocytes infected with adenovirus were placed together with the CFs in the lower layer. A sufficient amount of complete medium was added, and both types of cells were fully co-incubated through the pores of the membrane. After 24 h of co-culture, the cells were stimulated with 4 ng/ml TGF-β1 or vehicle, and then the 2 types of cells were harvested. The expression of p-Smad3 and BRCC3 in cardiomyocytes and CFs was detected by Western blot analysis.

### Human atrial adnexal tissue collection

Human sample collection was approved by the Affiliated Hospital of Yangzhou University Ethics Committee, and the study was conducted following the principles of Good Clinical Practice and the Declaration of Helsinki. Written consent was obtained from participants or their guardians. We collected atrial appendage tissue samples from 3 patients who had undergone heart valve replacement and had been diagnosed with both heart failure and valvular disease due to coronary heart disease, as well as from 3 control patients with heart valve replacement who had been diagnosed solely with valvular disease without concurrent heart failure.

### Statistical analysis

Data are expressed as mean ± standard error of the mean. Analyses were performed using SPSS 26.0 for Windows (SPSS Inc., Chicago, IL, USA). Heatmaps of gene expression were generated using a web-based tool (https://software.broadinstitute.org/morpheus/). The Shapiro–Wilk normality test was used initially to assess the normality of the distribution of the data. Comparisons between 2 groups involved the 2-tailed independent Student *t* test. Comparisons among more than 2 groups involved 1-way or 2-way analysis of variance with Bonferroni correction for multiple pairwise comparisons. *P* < 0.05 was considered statistically significant.

## Data Availability

The datasets used and/or analyzed in the current study are available from the corresponding author on reasonable request.

## References

[1] Fatehi Hassanabad A, Belke DD, Gordon PM, Teng G, Dundas JA, Zarzycki AN, Turnbull J, Deniset JF, Fedak PW. Pericardial fluid of patients with coronary artery disease can drive fibrosis via TGF-beta pathway. JACC Basic Transl Sci. 2024;9(11):1329–1344. 39619141 10.1016/j.jacbts.2024.06.007 PMC11604402

[2] Yang K, Zhao Y, Hu J, Gao R, Shi J, Wei X, Chen J, Hu K, Sun A, Ge J. ALKBH5 induces fibroblast-to-myofibroblast transformation during hypoxia to protect against cardiac rupture after myocardial infarction. J Adv Res. 2024;61:193–209. 37689242 10.1016/j.jare.2023.09.004 PMC11258655

[3] Frangogiannis NG. Cardiac fibrosis. Cardiovasc Res. 2021;117(6):1450–1488. 33135058 10.1093/cvr/cvaa324 PMC8152700

[4] Kong P, Shinde AV, Su Y, Russo I, Chen B, Saxena A, Conway SJ, Graff JM, Frangogiannis NG. Opposing actions of fibroblast and cardiomyocyte Smad3 signaling in the infarcted myocardium. Circulation. 2018;137(7):707–724. 29229611 10.1161/CIRCULATIONAHA.117.029622 PMC5812838

[5] Jin ZQ. MicroRNA targets and biomarker validation for diabetes-associated cardiac fibrosis. Pharmacol Res. 2021;174: Article 105941. 34656765 10.1016/j.phrs.2021.105941

[6] Huang S, Chen B, Su Y, Alex L, Humeres C, Shinde AV, Conway SJ, Frangogiannis NG. Distinct roles of myofibroblast-specific Smad2 and Smad3 signaling in repair and remodeling of the infarcted heart. J Mol Cell Cardiol. 2019;132:84–97. 31085202 10.1016/j.yjmcc.2019.05.006 PMC6579679

[7] Zhang X, Fang Y, Qin X, Zhang Y, Kang B, Zhong L, Liu B, Luo J, Wei Y. The role of MCPIP1 in macrophage polarization and cardiac function post-myocardial infarction. Adv Sci. 2025;12(25): Article e2500747. 10.1002/advs.202500747 PMC12225016 40285621

[8] Schilling SH, Datto MB, Wang XF. A phosphatase controls the fate of receptor-regulated Smads. Cell. 2006;125(5):838–840. 16751094 10.1016/j.cell.2006.05.015

[9] Tang LY, Yamashita M, Coussens NP, Tang Y, Wang X, Li C, Deng CX, Cheng SY, Zhang YE. Ablation of Smurf2 reveals an inhibition in TGF-β signalling through multiple mono-ubiquitination of Smad3. EMBO J. 2011;30(23):4777–4789. 22045334 10.1038/emboj.2011.393 PMC3243605

[10] Fukuchi M, Imamura T, Chiba T, Ebisawa T, Kawabata M, Tanaka K, Miyazono K. Ligand-dependent degradation of Smad3 by a ubiquitin ligase complex of ROC1 and associated proteins. Mol Biol Cell. 2001;12(5):1431–1443. 11359933 10.1091/mbc.12.5.1431 PMC34595

[11] Marx C, Held JM, Gibson BW, Benz CC. ErbB2 trafficking and degradation associated with K48 and K63 polyubiquitination. Cancer Res. 2010;70(9):3709–3717. 20406983 10.1158/0008-5472.CAN-09-3768 PMC2862137

[12] Song J, Zhang Y, Bai Y, Sun X, Lu Y, Guo Y, He Y, Gao M, Chi X, Heng BC, et al. The deubiquitinase OTUD1 suppresses secretory neutrophil polarization and ameliorates immunopathology of periodontitis. Adv Sci. 2023;10(30): Article e2303207. 10.1002/advs.202303207 PMC10602526 37639212

[13] Yao F, Zhou Z, Kim J, Hang Q, Xiao Z, Ton BN, Chang L, Liu N, Zeng L, Wang W, et al. SKP2- and OTUD1-regulated non-proteolytic ubiquitination of YAP promotes YAP nuclear localization and activity. Nat Commun. 2018;9(1):2269. 29891922 10.1038/s41467-018-04620-y PMC5995870

[14] Ha GH, Ji JH, Chae S, Park J, Kim S, Lee JK, Kim Y, Min S, Park JM, Kang TH, et al. Pellino1 regulates reversible ATM activation via NBS1 ubiquitination at DNA double-strand breaks. Nat Commun. 2019;10(1):1577. 30952868 10.1038/s41467-019-09641-9 PMC6450972

[15] Yan K, Ponnusamy M, Xin Y, Wang Q, Li P, Wang K. The role of K63-linked polyubiquitination in cardiac hypertrophy. J Cell Mol Med. 2018;22(10):4558–4567. 30102008 10.1111/jcmm.13669 PMC6156430

[16] Ng HM, Wei L, Lan L, Huen MS. The Lys63-deubiquitylating enzyme BRCC36 limits DNA break processing and repair. J Biol Chem. 2016;291(31):16197–16207. 27288411 10.1074/jbc.M116.731927 PMC4965568

[17] Shen H, Gao Y, Ge D, Tan M, Yin Q, Wei TW, He F, Lee TY, Li Z, Chen Y, et al. BRCC3 regulation of ALK2 in vascular smooth muscle cells: Implication in pulmonary hypertension. Circulation. 2024;150(2):132–150. 38557054 10.1161/CIRCULATIONAHA.123.066430 PMC11230848

[18] Shao G, Lilli DR, Patterson-Fortin J, Coleman KA, Morrissey DE, Greenberg RA. The Rap80-BRCC36 de-ubiquitinating enzyme complex antagonizes RNF8-Ubc13-dependent ubiquitination events at DNA double strand breaks. Proc Natl Acad Sci USA. 2009;106(9):3166–3171. 19202061 10.1073/pnas.0807485106 PMC2651241

[19] Xu M, Moresco JJ, Chang M, Mukim A, Smith D, Diedrich JK, Yates JR III, Jones KA. SHMT2 and the BRCC36/BRISC deubiquitinase regulate HIV-1 Tat K63-ubiquitylation and destruction by autophagy. PLoS Pathog. 2018;14(5): Article e1007071. 29791506 10.1371/journal.ppat.1007071 PMC5988312

[20] Yalcinkaya M, Liu W, Thomas LA, Olszewska M, Xiao T, Abramowicz S, Papapetrou EP, Westerterp M, Wang N, Tabas I, et al. BRCC3-mediated NLRP3 deubiquitylation promotes inflammasome activation and atherosclerosis in *Tet2* clonal hematopoiesis. Circulation. 2023;148(22):1764–1777. 37781816 10.1161/CIRCULATIONAHA.123.065344 PMC10872582

[21] Yan K, Li L, Wang X, Hong R, Zhang Y, Yang H, Lin M, Zhang S, He Q, Zheng D, et al. The deubiquitinating enzyme complex BRISC is required for proper mitotic spindle assembly in mammalian cells. J Cell Biol. 2015;210(2):209–224. 26195665 10.1083/jcb.201503039 PMC4508884

[22] Miskinyte S, Butler MG, Hervé D, Sarret C, Nicolino M, Petralia JD, Bergametti F, Arnould M, Pham VN, Gore AV, et al. Loss of BRCC3 deubiquitinating enzyme leads to abnormal angiogenesis and is associated with syndromic moyamoya. Am J Hum Genet. 2011;88(6):718–728. 21596366 10.1016/j.ajhg.2011.04.017 PMC3113251

[23] Gong K, Xing D, Li P, Hilgers RH, Hage FG, Oparil S, Chen YF. cGMP inhibits TGF-β signaling by sequestering Smad3 with cytosolic β2-tubulin in pulmonary artery smooth muscle cells. Mol Endocrinol. 2011;25(10):1794–1803. 21868450 10.1210/me.2011-1009 PMC3182417

[24] Cilenti L, Balakrishnan MP, Wang XL, Ambivero C, Sterlicchi M, del Monte F, Ma XL, Zervos AS. Regulation of Abro1/KIAA0157 during myocardial infarction and cell death reveals a novel cardioprotective mechanism for Lys63-specific deubiquitination. J Mol Cell Cardiol. 2011;50(4):652–661. 21195082 10.1016/j.yjmcc.2010.12.015 PMC3057332

[25] Calcagno DM, Taghdiri N, Ninh VK, Mesfin JM, Toomu A, Sehgal R, Lee J, Liang Y, Duran JM, Adler E, et al. Single-cell and spatial transcriptomics of the infarcted heart define the dynamic onset of the border zone in response to mechanical destabilization. Nat Cardiovasc Res. 2022;1(11):1039–1055. 39086770 10.1038/s44161-022-00160-3 PMC11290420

[26] Xu GR, Zhang C, Yang HX, Sun JH, Zhang Y, Yao TT, Li Y, Ruan L, An R, Li AY. Modified citrus pectin ameliorates myocardial fibrosis and inflammation via suppressing galectin-3 and TLR4/MyD88/NF-κB signaling pathway. Biomed Pharmacother. 2020;126: Article 110071. 32172066 10.1016/j.biopha.2020.110071

[27] Chen C, Wang J, Hou C, Lian W, Zhu X, Hu J, Liu C. Bushen Huoxue Yiqi formula alleviates cardiac fibrosis in ischemic heart failure through SIRT1/Notch1 pathway-mediated EndMT. Phytomedicine. 2024;135: Article 156252. 39566406 10.1016/j.phymed.2024.156252

[28] Chen X, Li X, Wu X, Ding Y, Li Y, Zhou G, Wei Y, Chen S, Lu X, Xu J, et al. Integrin beta-like 1 mediates fibroblast–cardiomyocyte crosstalk to promote cardiac fibrosis and hypertrophy. Cardiovasc Res. 2023;119(10):1928–1941. 37395147 10.1093/cvr/cvad104

[29] Zeqiraj E, Tian L, Piggott CA, Pillon MC, Duffy NM, Ceccarelli DF, Keszei AF, Lorenzen K, Kurinov I, Orlicky S, et al. Higher-order assembly of BRCC36–KIAA0157 is required for DUB activity and biological function. Mol Cell. 2015;59(6):970–983. 26344097 10.1016/j.molcel.2015.07.028 PMC4579573

[30] Narasimhan A, Min SH, Johnson LL, Roehrich H, Cho W, Her TK, Windschitl C, O’Kelly RD, Angelini L, Yousefzadeh MJ, et al. The *Ercc1*^−/Δ^ mouse model of XFE progeroid syndrome undergoes accelerated retinal degeneration. Aging Cell. 2025;24(3): Article e14419. 39604117 10.1111/acel.14419 PMC11896507

[31] Nakamura K, Kustatscher G, Alabert C, Hödl M, Forne I, Voelker-Albert M, Satpathy S, Beyer TE, Mailand N, Choudhary C, et al. Proteome dynamics at broken replication forks reveal a distinct ATM-directed repair response suppressing DNA double-strand break ubiquitination. Mol Cell. 2021;81(5):1084–99 e6. 33450211 10.1016/j.molcel.2020.12.025 PMC7939521

[32] Gong S, Zhai M, Shi J, Yu G, Lei Z, Shi Y, et al. TREM2 macrophage promotes cardiac repair in myocardial infarction by reprogramming metabolism via SLC25A53. Cell Death Differ. 2024;31(2):239–253. 38182899 10.1038/s41418-023-01252-8 PMC10850484

[33] Gu L, Wang S, Zhou L, Wang W, Bao Y, He Y, Yang T, Sun J, Jiang Q, Shan T, et al. Targeting NLRC5 in cardiomyocytes protects postinfarction cardiac injury by enhancing autophagy flux through the CAVIN1/CAV1 axis. Commun Biol. 2025;8(1):292. 39988583 10.1038/s42003-025-07755-z PMC11847941

[34] Lucas JA, Zhang Y, Li P, Gong K, Miller AP, Hassan E, Hage F, Xing D, Wells B, Oparil S, et al. Inhibition of transforming growth factor-βsignaling induces left ventricular dilation and dysfunction in the pressure-overloaded heart. Am J Physiol Heart Circ Physiol. 2010;298(2):H424–H432. 19933419 10.1152/ajpheart.00529.2009 PMC2822586

[35] Subramani K, Bander J, Chen S, Suárez-Fariñas M, Venkatesan T, Subrahmanian S, Varshney R, Kini A, Sharma S, Rifkin DB, et al. Evidence that anemia accelerates AS progression via shear-induced TGF-β1 activation: Heyde’s syndrome comes full circle. JACC Basic Transl Sci. 2024;9(2):185–199. 38510715 10.1016/j.jacbts.2023.09.007 PMC10950403

[36] Zhang J, Sheng J, Dong L, Xu Y, Yu L, Liu Y, Huang X, Wan S, Lan HY, Wang H. Cardiomyocyte-specific loss of RNA polymerase II subunit 5-mediating protein causes myocardial dysfunction and heart failure. Cardiovasc Res. 2019;115(11):1617–1628. 30590389 10.1093/cvr/cvy307

[37] Umbarkar P, Singh AP, Gupte M, Verma VK, Galindo CL, Guo Y, Zhang Q, McNamara JW, Force T, Lal H. Cardiomyocyte SMAD4-dependent TGF-β signaling is essential to maintain adult heart homeostasis. JACC Basic Transl Sci. 2019;4(1):41–53. 30847418 10.1016/j.jacbts.2018.10.003 PMC6390466

[38] Bugyei-Twum A, Ford C, Civitarese R, Seegobin J, Advani SL, Desjardins JF, Kabir G, Zhang Y, Mitchell M, Switzer J, et al. Sirtuin 1 activation attenuates cardiac fibrosis in a rodent pressure overload model by modifying Smad2/3 transactivation. Cardiovasc Res. 2018;114(12):1629–1641. 29800064 10.1093/cvr/cvy131 PMC6148332

[39] Dobaczewski M, Bujak M, Li N, Gonzalez-Quesada C, Mendoza LH, Wang XF, Frangogiannis NG. Smad3 signaling critically regulates fibroblast phenotype and function in healing myocardial infarction. Circ Res. 2010;107(3):418–428. 20522804 10.1161/CIRCRESAHA.109.216101 PMC2917472

[40] Gao S, Alarcón C, Sapkota G, Rahman S, Chen PY, Goerner N, Macias MJ, Erdjument-Bromage H, Tempst P, Massagué J. Ubiquitin ligase Nedd4L targets activated Smad2/3 to limit TGF-β signaling. Mol Cell. 2009;36(3):457–468. 19917253 10.1016/j.molcel.2009.09.043 PMC2796330

[41] Inui M, Manfrin A, Mamidi A, Martello G, Morsut L, Soligo S, Enzo E, Moro S, Polo S, Dupont S, et al. USP15 is a deubiquitylating enzyme for receptor-activated SMADs. Nat Cell Biol. 2011;13(11):1368–1375. 21947082 10.1038/ncb2346

[42] Balakirev MY, Tcherniuk SO, Jaquinod M, Chroboczek J. Otubains: A new family of cysteine proteases in the ubiquitin pathway. EMBO Rep. 2003;4(5):517–522. 12704427 10.1038/sj.embor.embor824 PMC1319179

[43] Herhaus L, Al-Salihi M, Macartney T, Weidlich S, Sapkota GP. OTUB1 enhances TGFβ signalling by inhibiting the ubiquitylation and degradation of active SMAD2/3. Nat Commun. 2013;4:2519. 24071738 10.1038/ncomms3519 PMC3791481

[44] Wiener R, Zhang X, Wang T, Wolberger C. The mechanism of OTUB1-mediated inhibition of ubiquitination. Nature. 2012;483(7391):618–622. 22367539 10.1038/nature10911 PMC3319311

[45] Huang Z, Shen S, Wang M, Li W, Wu G, Huang W, Luo W, Liang G. Mouse endothelial OTUD1 promotes angiotensin II-induced vascular remodeling by deubiquitinating SMAD3. EMBO Rep. 2023;24(3): Article e56135. 36579465 10.15252/embr.202256135 PMC9986815

[46] Su SA, Zhang Y, Li W, Xi Y, Lu Y, Shen J, Ma Y, Wang Y, Shen Y, Xie L, et al. Cardiac Piezo1 exacerbates lethal ventricular arrhythmogenesis by linking mechanical stress with Ca^2+^ handling after myocardial infarction. Research. 2023;6:0165. 37303604 10.34133/research.0165 PMC10255393

[47] Voorrips SN, Palm CL, Saucedo-Orozco H, Mahmoud B, Schouten EM, Feringa AM, Sanchez-Aguilera PI, Nijholt KT, Yurista SR, van Der Meer P, et al. Myocardial ketone body oxidation contributes to empagliflozin-induced improvements in cardiac contractility in murine heart failure. Eur J Heart Fail. 2025;27(7):1353–1358. 40069113 10.1002/ejhf.3633 PMC12370586

[48] Mok MT, Henderson BR. Three-dimensional imaging reveals the spatial separation of γH2AX–MDC1–53BP1 and RNF8–RNF168–BRCA1-A complexes at ionizing radiation-induced foci. Radiother Oncol. 2012;103(3):415–420. 22633816 10.1016/j.radonc.2012.04.009

[49] Shukla PC, Singh KK, Quan A, Al-Omran M, Teoh H, Lovren F, Cao L, Rovira II, Pan Y, Brezden-Masley C, et al. BRCA1 is an essential regulator of heart function and survival following myocardial infarction. Nat Commun. 2011;2:593. 22186889 10.1038/ncomms1601 PMC3247816

[50] Singh KK, Shukla PC, Quan A, Desjardins JF, Lovren F, Pan Y, Garg V, Gosal S, Garg A, Szmitko PE, et al. BRCA2 protein deficiency exaggerates doxorubicin-induced cardiomyocyte apoptosis and cardiac failure. J Biol Chem. 2012;287(9):6604–6614. 22157755 10.1074/jbc.M111.292664 PMC3325595

[51] Rabl J, Bunker RD, Schenk AD, Cavadini S, Gill ME, Abdulrahman W, Andrés-Pons A, Luijsterburg MS, Ibrahim AFM, Branigan E, et al. Structural basis of BRCC36 function in DNA repair and immune regulation. Mol Cell. 2019;75(3):483–97 e9. 31253574 10.1016/j.molcel.2019.06.002 PMC6695476

[52] Oh J, Symington LS. Role of the Mre11 complex in preserving genome integrity. Genes (Basel). 2018;9(12). 10.3390/genes9120589 PMC6315862 30501098

[53] Dubrovska A, Kanamoto T, Lomnytska M, Heldin CH, Volodko N, Souchelnytskyi S. TGF*β*1/Smad3 counteracts BRCA1-dependent repair of DNA damage. Oncogene. 2005;24(14):2289–2297. 15735739 10.1038/sj.onc.1208443

[54] Cheedipudi SM, Asghar S, Marian AJ. Genetic ablation of the DNA damage response pathway attenuates lamin-associated dilated cardiomyopathy in mice. JACC Basic Transl Sci. 2022;7(12):1232–1245. 36644279 10.1016/j.jacbts.2022.06.015 PMC9831927

[55] Ye B, Hou N, Xiao L, Xu Y, Xu H, Li F. Dynamic monitoring of oxidative DNA double-strand break and repair in cardiomyocytes. Cardiovasc Pathol. 2016;25(2):93–100. 26764141 10.1016/j.carpath.2015.10.010 PMC4801000

[56] Martinez Lagunas K, Savcigil DP, Zrilic M, Carvajal Fraile C, Craxton A, Self E, Uranga-Murillo I, de Miguel D, Arias M, Willenborg S, et al. Cleavage of cFLIP restrains cell death during viral infection and tissue injury and favors tissue repair. Sci Adv. 2023;9(30): Article eadg2829. 37494451 10.1126/sciadv.adg2829 PMC10371024

[57] Wang Z, Niu K, Liu W, Wang X, Yang B, Li T, Chen Y, Jin Y, Chen Y, Lin Y, et al. ER-phagy activation by AMFR attenuates cardiac fibrosis post-myocardial infarction via mTORC1 pathway. Adv Sci (Weinh). 2025;12(37): Article e04552. 40673870 10.1002/advs.202504552 PMC12499418

[58] Bei Y, Wang H, Liu Y, Su Z, Li X, Zhu Y, Zhang Z, Yin M, Chen C, Li L, et al. Exercise-induced miR-210 promotes cardiomyocyte proliferation and survival and mediates exercise-induced cardiac protection against ischemia/reperfusion injury. Research. 2024;7:0327. 38410280 10.34133/research.0327 PMC10895486

[59] Shan TK, Yang TT, Jing P, Bao YL, Zhou LH, Zhu T, Shi XY, Wei TW, Wang SB, Gu LF, et al. Circular RNA IGF1R promotes cardiac repair via activating β-catenin signaling by interacting with DDX5 in mice after ischemic insults. Research. 2024;7:0451. 39193132 10.34133/research.0451 PMC11347128

[60] Zhang H, Liu L, Shen C, Jiang X, Liu J, Chen J, Xu S, Mo Y. Lactate-induced mitochondrial calcium uptake 3 aggravates myocardial ischemia-reperfusion injury by promoting neutrophil extracellular trap formation. Research. 2025;8:0705. 40452820 10.34133/research.0705 PMC12123085

[61] Xu DS, Wang Y, Li ZR, Mo PL, Chen MT, Zhao RZ, Fan Z, Mo WJ, Li QL, Pi FC, et al. Shenxiong Yixin decoction polysaccharides exert therapeutic effects against myocardial ischemia-reperfusion injury via intestinal exosomal microRNA-21 regulation. Pharmacol Res. 2025;215: Article 107725. 40209966 10.1016/j.phrs.2025.107725

