# Supplementary material for: Therapeutic Potential of the Deubiquitinase BRCC3 in Attenuating Ischemic Myocardial Injury and Ventricular Remodeling
Source: Research (Wash D C). 2026 Sep 3;9:1418. doi: 10.34133/research.1418 (PMC13538938; doi:10.34133/research.1418)
Supplement: Supplementary file 1 [file research.1418.f1.docx]

**Therapeutic Potential of Deubiquitinase BRCC3 in Attenuating Ischemic Myocardial Injury and Ventricular Remodeling**.

Deshan Yao^1,#^, Sichuan Wang ^1,#^, Rujun Li^#1^, Xin Pan^1^, Zixuan Chen^1^, Mengxuan Yuan^1^, Pei Zhao ^1^, Zhengang Zhang^1^, Kaizheng Gong^1,*^

^1^Department of Cardiology, Affiliated Hospital of Yangzhou University, Yangzhou University, Institute of Cardiovascular Disease, Yangzhou Key Lab of Innovation Frontiers in Cardiovascular Disease, Yangzhou, Jiangsu, 225001, China;

^#^ These authors contributed equally to this work

^*^ This author jointly supervised this work

The authors have declared that no conflict of interest exists.

Correspondence to:

Kaizheng Gong, MD, PhD; Email: yungkzh@163.com. Department of Cardiology, Affiliated Hospital of Yangzhou University, Yangzhou University. No.368 Hanjiang Middle Rd, Yangzhou 225001, China;

SUPPLEMENTARY MATERIALS

Supplemental Figures

**Supplemental Figure S1: Generation of cardiac-specific *Brcc*3 knockout mice.** **A** Diagram of the conditional knockout allele construct used for the generation of *Brcc3*-KO mice using Cre-LoxP technology. The *Brcc3* gene has eleven exons. Exon 5 was selected as the conditional knockout region since its deletion results in the loss of function of the mouse *Brcc3* gene. LoxP sites were cloned into the *Brcc3* gene via Flp recombination flanking exons 5 in the CKO region. After Cre-mediated recombination, Cre-mediated excision of exons 5 resulted in a frame shift mutation from exon 4 to 6, leading to an early translational stop and subsequent conditional *Brcc3* knock out (*Brcc3*-KO). **B** *Upper* lanes: genotyping with PCR from mice using primers targeting *Brcc3*. *Brcc3^flox+/+^*mice had only one 485bp band while *Brcc3^flox+/-^* mice had two bands of 485bp and 357bp. Non-transgenic wild type C57BL/6 had only one 357bp band. *Lower* lanes: genotyping with PCR from mice using primers targeting Cre. *Brcc3*-KO (lanes 1 and 3) and littermate control mice (*Brcc3^flox+/+, Cre-^*, lane 6, B3fl/fl) were used in this study. **C** Expression of BRCC3 in various tissues, including left ventricles, from *Brcc3-*KO and B3-fl-fl mice determined by Western blot analysis using anti-BRCC3 antibody. β-actin as a loading control, n=4 mice. Results are expressed as mean ± SEM, Statistical analysis: student’s t tests.

**Figure S2**: **Measurements of infarction size and left ventricular structure and function in *Brcc3*-KO mice with AMI***.* **A, B** Representative TTC-stained myocardial infarction areas were shown in *Brcc3*-KO and B3-fl/fl groups on day 7 after surgery (n=5-8). Data are mean ± SEM (one-way ANOVA followed by Bonferroni multiple comparison tests).

**Figure S3:** **BRCC3 affects paracrine signaling in cardiac muscle cells.** In primary cultured cardiomyocytes, adenoviruses carrying empty vectors and adenoviruses overexpressing BRCC3 were transfected. The cardiomyocytes were co-cultured with fibroblasts. The culture medium was collected at 1 hour, 24 hours, and after TGF-β1 stimulation for 0.5 hours. The expression levels of TGF-β1 (**A**), IL-6 (**B**), and extracellular vesicle milk fat globule-epidermal growth factor 8 (MFGE8) (**C**) in the culture medium were detected by ELISA. n=6. Results are expressed as mean ± SEM, Statistical analysis: One-way ANOVA followed by Bonferroni multiple comparison tests.

**Figure S4**: **Smad3 mediates the effect of BRCC3 on the inhibition of TGF-β.** **A** Isolated cardiac fibroblasts were infected with AD-Ctrl or AD-*Brcc3* for 72 hours and pre-treatment with a specific Smad3 inhbitor (E)-SIS3 (10uM) for 30 min, and then were stimulated with TGF-β1 (4ng/ml) or vehicle (Veh.) for 12 hours. mRNA levels of connective tissue growth factor (*CTGF)* and *Periostin* were determined by real-time reverse transcription‑PCR (RT-PCR) analysis. Glyceraldehyde-3-phosphate dehydrogenase (GAPDH) was an internal control. Results are expressed as mean ± SEM, Statistical analysis: One-way ANOVA followed by Bonferroni multiple comparison tests. N.S., non-significance. **B** Isolated cardiac fibroblasts were infected with AD-Ctrl or AD-*Brcc3* for 72 hours and were stimulated with TGF-β1 (4ng/ml) or vehicle (Veh.) for 30 minutes. Level of smad2 phosphorylation was detected by Western blot analysis by using a specific antibody against Smad2. The experiments were repeated, and representative results are shown.

**Figure S5:** **BRCC3 inhibits the activation of the TGF-β/Smad3 signaling pathway depending on its deubiquitinating enzyme activity**. Primary cultured rat cardiomyocytes were transfected with empty vector adenovirus (AD-Ctrl), adenovirus-carrying *Brcc3* (AD-*Brcc3*), and adenovirus-carrying mutant *Brcc3* (AD-*Brcc3* mut) for 3 days and then treated with 4 ng/ml TGF-β1 or Vehicle (Veh.). **A** Western blot was used to detect the expression of p-Smad3 and Smad3 in cardiomyocytes, with glyceraldehyde-3-phosphate dehydrogenase (GAPDH) as the internal control, n = 6. **B** Real-time reverse transcription‑PCR was used to detect the expression of connective tissue growth factor (*CTGF)*, *Collagen I*, and *Collagen III* mRNA in cardiac muscle cells, with GAPDH as the internal reference, n=12. Results are expressed as mean ± SEM, Statistical analysis: One-way ANOVA followed by Bonferroni multiple comparison tests.

.

**Figure S6**: **Effect of BRCC3 on K48-, K63-linked polyubiquitination of proteins.** **A** Isolated cardiac fibroblasts were infected Ad-null or Ad-*Brcc3* for 72 hours and treated with proteasome inhibitor MG132 or vehicle. Level of K48-linded polyubiquitination of Smad3 was determined by immunoprecipitation study using antibodies against Smad3 or K48-ub. ɑ-tubulin served as an input control. The experiments were repeated, and representative results are shown. **B** Primary cultured cardiomyocytes were stimulated with 4 ng/ml TGF-β1 for 0, 5, 10, 15, and 30 minutes. Smad3 antibody was used for co-immunoprecipitation, K63-UB antibody was used to detect the ubiquitination modification level at the K63 site of Smad3, and parallel detection of p-Smad3 expression was conducted. Co-IP detected the ubiquitination modification level of Smad3 at the K63 site in cardiomyocytes, and western blot detected the expression of p-Smad3. Co-IP experiment detects the ubiquitination modification level of Smad3 at K63 site in myocardial tissue. **C** WT and *Brcc3-*Tg mice underwent sham or left anterior descending branch ligation, and heart tissue protein was extracted and total K63-linked polyubiquitination levels were determined by Western blot analysis by using anti-body against K63-Ub. **D** Co-IP experiment detects the ubiquitination modification level of Smad3 at K63 site in myocardial tissue. Data are mean ± SEM, n=4 mice/group (one-way ANOVA followed by Bonferroni multiple comparison tests).

**Figure S7**: **Predicted binding mode of** **BRCC3 and SMAD3-MH2 domain**. **A, B** Structures of BRCC3 (PDB ID: 6R8F) and SMAD3-MH2 (PDB ID: 5XOC) from PDB Database. SMAD3-MH2 contains five α-helices (violet) and eleven β-sheets (green). Surfaces are color coded according to electrostatic potential: blue, positive areas; red, negative areas. **C** Predicted binding mode of BRCC3 and SMAD3-MH2 by ZDOCK server. The negative areas of BRCC3 binds with the positive areas of SMAD3-MH2 through electrostatic interactions. **D** A schematic diagram showing binding between BRCC3 (tan) and SMAD3-MH2 (sky blue) from different angles.

**Figure S8. BRCC3 regulates pathological injury and fibrotic responses specifically through the Smad3 K333 residue. A** Cardiomyocytes were infected with adenovirus encoding short hairpin RNA (shRNA) targeting BRCC3 (AD-*Brcc3* shRNA) or control shRNA (Ad-Ctrl), and co-transfected with either wild-type (WT) Smad3 or the K333R mutant adenovirus. Cells were then subjected to oxygen–glucose deprivation (OGD) stimulation to model myocardial injury. Representative immunoblots and quantitative densitometric analysis of phosphorylated Smad3 (p-Smad3) and total Smad3 in cardiomyocytes. Glyceraldehyde-3-phosphate dehydrogenase (GAPDH) was a loading control. n=6. **B** ELISA method to detect myocardial cells of creatine kinase isoenzyme CK-MB and malondialdehyde MDA expression level. n=6. **C** Cardiac fibroblasts were infected with adenovirus encoding shRNA targeting BRCC3 (AD-*Brcc3* shRNA) or control shRNA (Ad-Ctrl), and co-transfected with either wild-type (WT) Smad3 or the K333R mutant adenovirus. Cells were then subjected to TGF-β1 stimulation to model myocardial injury. Representative immunoblots and quantitative densitometric analysis of phosphorylated Smad3 (p-Smad3) and total Smad3 in CFs. GADPH was a loading control. n=6. **D** Real-time reverse transcription‑PCR was used to detect the expression levels of extracellular matrix Collagen I and Collagen III mRNA in cardiac fibroblasts. GAPDH was an internal control, n=6. Results are expressed as mean ± SEM, Statistical analysis: One-way ANOVA followed by Bonferroni multiple comparison tests. n.s., non-significance.

**Figure. S9. TGFβ/Smad3 inhibitor alleviates myocardial remodeling and cardiac function in myocardial infarction-induced myocardial-specific *Brcc3* knockout mice.** Mice were treated with 100mg/kg of TGF-β1/Smad3 signaling pathway inhibitor IN-1 by gavage for 3 days, and then a myocardial infarction model was constructed using the left anterior descending artery method. The hearts of mice were examined for structure and function 7 days after the operation. **A** Echocardiography detected LVESD, LVEDD, LVEF, and LVFS of mice in each group, IN-1: TGF-β1/Smad3 inhibitor, n = 5. **B** Representative light microscopic images of 40x magnification of heart sections stained with Masson's method, and the values of myocardial fiber volume fraction (CVF) in each group of mice after myocardial infarction. bar=500µm, n = 5. **C** Western blot analysis for left ventricle levels of p-Smad3 and Smad3 protein expression. Glyceraldehyde-3-phosphate dehydrogenase (GAPDH) was a loading control. Results are expressed as mean ± SEM, Statistical analysis: One-way ANOVA followed by Bonferroni multiple comparison tests.

**Figure 10: BRCC3 protects cardiomyocytes against sustained TGF-β1 and H_2_O_2_ challenges.** Mouse cardiomyocytes were infected AD-Ctrl or AD-*Brcc3* for 72 hours. **A** Cells were pretreated with a specific inhibitor of Smad3, (E)-SIS3 (10uM) for 30 minutes and then treated with 50ng/ml TGF-β1 or vehicle (Veh) for 48 hours. Western blot analysis of DSB formation markers (i.e γH2AX and 53BP1). β-actin was a loading control. The experiments were repeated, and representative results are shown. **B** Cardiomyocytes infected with AD-*Brcc3* or AD-Ctrl was challenged with H_2_O_2_ (200 uM) for 1 hour. Representative immunofluorescence staining (400×magnification) of cardiomyocytes for assessment of DSB formation and DNA damage repair with anti-γH2AX or anti-RAD51 antibodies. Nuclei were stained with DAPI (Blue). γH2AX and RAD51 foci formation were quantitatively analyzed. **C** Representative western blot analysis of phosphorylated ATM, ATM, γH2AX, and RAD51. β-actin was a loading control. Results are expressed as mean ± SEM, Statistical analysis: One-way ANOVA followed by Bonferroni multiple comparison tests.

**Table S1 Cardiac function indexes of patients with coronary artery disease and normal cardiac function (Ctrl) or heart failure (HF)**

| Patients | Samples | Age (years) | Sex | LVEF (%) | LVFS (%) | BNP (pg/ml) |
| --- | --- | --- | --- | --- | --- | --- |
| Ctrl | 1 | 80 | Male | 62 | 34 | 321 |
|  | 2 | 65 | Male | 72 | 41 | 84 |
|  | 3 | 70 | Male | 70 | 40 | 164 |
| HF | 4 | 72 | Female | 30 | 17 | 2581 |
|  | 5 | 69 | Male | 36 | 18 | 1586 |
|  | 6 | 71 | Male | 26 | 16 | 2190 |

BNP: B-type natriuretic peptide; LVEF: left ventricular ejection fraction; LVFS: left ventricular fractional shortening;
